# First total synthesis of hoshinoamide A

**DOI:** 10.3762/bjoc.17.201

**Published:** 2021-12-15

**Authors:** Haipin Zhou, Zihan Rui, Yiming Yang, Shengtao Xu, Yutian Shao, Long Liu

**Affiliations:** 1 College of Materials & Chemical Engineering, Chuzhou University, Chuzhou 239000, China; 2 State Key Laboratory of Natural Medicines and Department of Medicinal Chemistry, China Pharmaceutical University,24 Tong Jia Xiang, Nanjing 210009, China; 3 Taizhou Medical Hi-Tech Development Public Services Platform, Taizhou 225300, China

**Keywords:** antimalarial, highly methylated polypeptides, hoshinoamides, total synthesis

## Abstract

Hoshinoamides A, B and C, linear lipopeptides, were isolated from the marine cyanobacterium *Caldora penicillata*, with potent antiplasmodial activity against chloroquine-sensitive *Plasmodium falciparum*. Herein, we describe the first total synthesis of hoshinoamide A by the combination of liquid-phase and solid-phase peptide synthesis. Liquid-phase synthesis is to improve the coupling yield of ʟ-Val^3^ and *N*-Me-ᴅ-Phe^2^. Connecting other amino acids efficiency and convergence is achieved by solid-state synthesis. Our synthetic strategy could synthesize the target peptide in high yield with good purity

## Introduction

Malaria is an insect-borne infectious disease caused by parasites of the genus *Plasmodium*, which seriously threatens human life and health [1]. Half of the world's population is at the risk of malaria, causing 200 million new infections and killing hundreds of thousands of people each year [2]. Current medicines for malaria include quinolone [3–4], folic acid antagonist [5–6] and artemisinin derivatives [7]. The emergence of drug resistance makes the efficacy of these drugs decline year by year, forcing scientists to constantly search for new antimalarial drugs [8–10].

**Figure 1:**
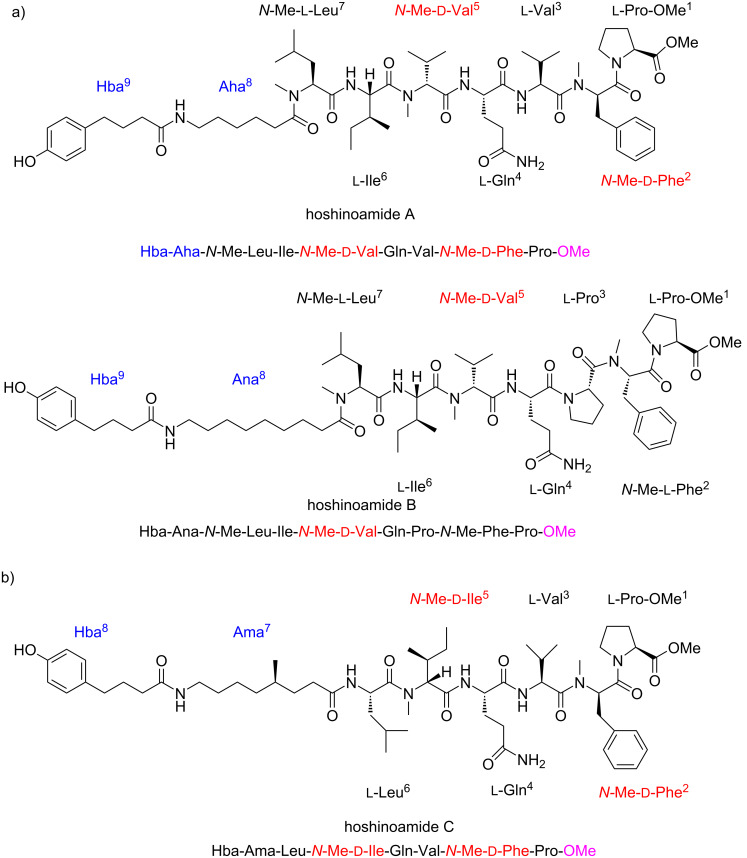
a) Structures of hoshinoamides A and B. b) Structure of hoshinoamide C.

In recent years, Iwasaki and co-workers have reported three novel linear lipopeptide natural products, hoshinoamides A, B [11] and C [12], from a microbial metabolite of marine cyanobacterium *Caldora penicillata* (Figure 1). Hoshinoamides A and B showed potent activities against chloroquine-sensitive *Plasmodium falciparum* 3D7 with IC_50_ values of 0.52 and 1.0 μM, respectively. Hoshinoamide C inhibited the growth of the malaria parasites (IC_50_ = 0.96 μM) and African sleeping sickness (IC_50_ = 2.9 μM). Hoshinoamide C was firstly synthesized from Boc-protected amino acids in liquid phase [12]. Both hoshinoamides A and B are highly methylated polypeptides containing three *N*-methyl amino acids: *N*-Me-ʟ-Leu^7^, *N*-Me-ᴅ-Val^5^ and *N*-Me-ᴅ/ʟ-Phe^2^. Hoshinoamide C includes two *N*-methyl amino acids: *N*-Me-ᴅ-Phe^2^ and *N*-Me-ᴅ-IIe^5^. The C-terminal Pro is functionalized as methyl ester, while the *N*-terminal polypeptide is linked to the long alkyl chain amino acid Aha^8^/Ana^8^/Ama^7^ and *p*-hydroxybenzoic acid Hba^9^/Hba^8^. Hoshinoamides have a relatively simple structure and therefore make an attractive target for further medicinal chemistry studies. To enable these new SAR studies, we need to develop an efficient synthetic method to provide sufficient materials firstly. Hoshinoamide A shows a better antimalarial activity and less cytotoxicity compared to hoshinoamide B. Herein, we report the initial progress on the total synthesis of hoshinomaide A.

The key challenges for the total synthesis of hoshinoamide A are the coupling of highly methylated amino acids and the purification of hydrophobic peptides.

## Results and Discussion

**Scheme 1:**
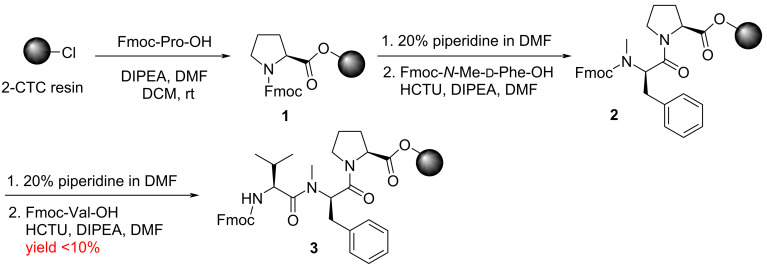
Synthesis of resin-bound tripeptide 3 by SPPS. DIPEA: *N*,*N*-diisopropylethylamine; HCTU: *O*-(6-chloro-1-hydrocibenzotriazol-1-yl)-1,1,3,3-tetramethyluronium hexafluorophosphat.

As shown in Scheme 1, we initially tested Fmoc solid-phase peptide synthesis (SPPS) [13] to get 2-chlorotrityl resin-bound Pro^1^-(*N*-Me)-Phe^2^ dipeptide **2** under the conditions of HCTU and DIPEA. Unfortunately, the *N*-Me coupling proceeded in low yield (<10%).

**Scheme 2:**
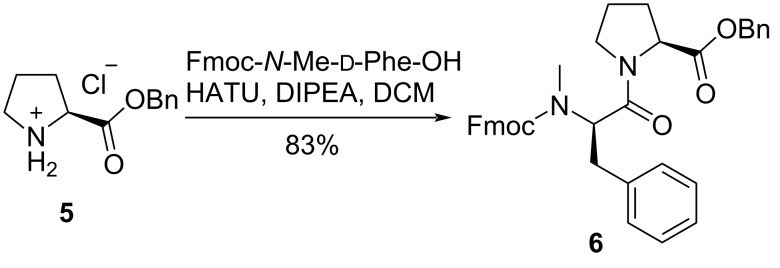
Synthesis of dipeptide **6**. HATU: 2-(7-azabenzotriazol-1-yl)-*N*,*N*,*N*',*N*'-tetramethyluronium hexafluorophosphate.

In order to improve the coupling yield of the hindered peptide, we tried the condensation of Val^3^ with the dipeptide in solution. Firstly, Pro-OBn **5** was coupled with Fmoc-*N*-Me-ᴅ-Phe-OH by the treatment of HATU and DIPEA [14], giving dipeptide **6** in 83% yield (Scheme 2).

In principle, the coupling of **6** and Fmoc-Val-OH under suitable conditions would deliver tripeptide **7**. Keeping this in mind, we next screened a series of coupling reagents [15]. As shown in Table 1, the coupling reagents have a significant effect on the efficiency of the reactions. At the very beginning, we selected HCTU and DIPEA as the coupling reagent (Table 1, entry 1). The desired tripeptide **7** was obtained in 36% yield. Similar conditions were selected by Iwasaki’s group during the total synthesis of hoshinoamide C. To our delight, when HATU was used, the overall yield of tripeptide **7** was rised to more than 70% (Table 1, entries 2 and 3). Tripeptide **7** was produced in 78% isolated yield when HATU/DIPEA as the coupling reagent was used (Table 1, entry 2). Only trace product could be detected with EEDQ (Table 1, entry 4). Further screening did not give better results (Table 1, entries 5 and 6). The purity of tripeptide **7** was determined by HPLC and no racemization was observed, ensuring the smooth progress of the total synthesis of hoshinoamide A.

**Table 1:** Hindered peptide coupling: conditions and yields.

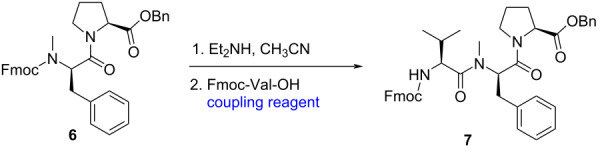			

Entry	Coupling reagent^a^	Yield^b^ (%)	Racemization^c^

1	HCTU, DIPEA	36	NO
2	HATU, DIPEA	78^d^	NO
3	HATU, HOAT^e^, DIPEA	75	NO
4	EEDQ^f^	trace	NO
5	DIC^g^, HOAT	41	NO
6	DIC, Oxyma^h^	24	NO

^a^0.1 mmol/L of dipeptide **6** and Fmoc-Val in DMF, 1.5 equiv coupling reagent, rt, 3 h. ^b^Yield was determined by ^1^H NMR data analysis. ^c^Racemization was determined by HPLC. ^d^Isolated yield. ^e^HOAT: 3*H*-[1,2,3]-triazolo[4,5-*b*]pyridin-3-ol; ^f^EEDQ: 2-ethoxy-1-ethoxycarbonyl-1,2-dihydroquinoline; ^g^DIC: *N*,*N*'-diisopropylcarbodiimide; ^h^Oxyma: 17-(acetyloxy)-3-methoxy-20-oxopregna-3,5-diene-6-carboxaldehyde.

**Scheme 3:**
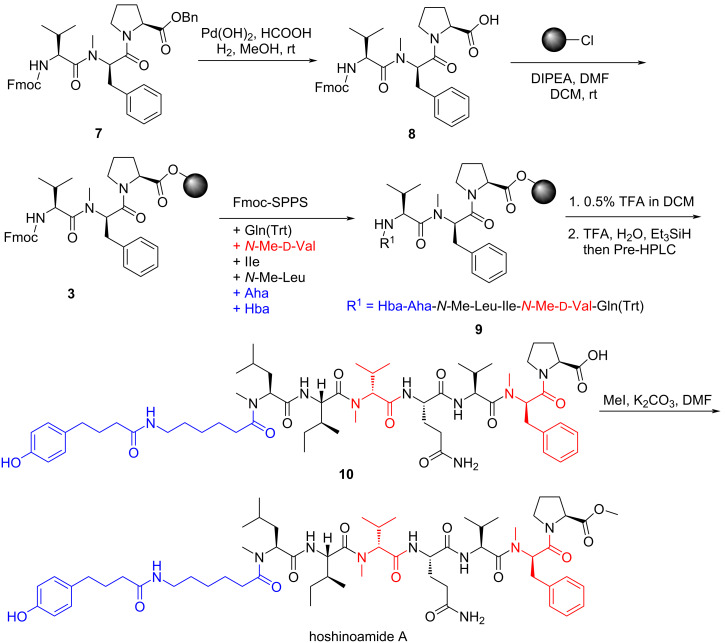
Synthesis of hoshinoamide A.

With the tripeptide **7** in hand, we went on to construct the peptide scaffold (Scheme 3). When tripeptide **7** was subjected to Pd-catalyzed hydrogenation conditions [16], the benzyl group was selectively cleaved to generate **8**. Treatment of 2-chlorotrityl chloride (CTC) resin with **8** (4 equiv) and DIPEA successfully produced the resin-bound tripeptide **3** in good yield [17]. It should be noted that unreacted **8** can be largely recovered by a quick silica gel chromatography. The N terminus of **3** was then sequentially extended with properly protected ʟ-Gln^4^, *N*-Me-ᴅ-Val^5^, ʟ-Ile^6^, *N*-Me- ʟ-Leu^7^, Ana^8^, and Hba^9^ units to give 9-mer peptide **9** using the standard SPPS procedure [13]. When ʟ-Ile^6^ and Ana^8^ were coupled, the reaction time was prolonged to 3 h in order to increase the yield. The peptide chain was then cleaved from resin by the solution of 0.5% TFA in DCM. The Trt group was removed with aqueous solution of TFA to give 9-mer peptide **10** in good yield. The carboxylic acid of 9-mer peptide **10** was converted to the methyl ester with MeI and K_2_CO_3_ in DMF [18], delivering the final natural product hoshinoamide A in 2% yield (10 mg). The spectroscopic data of synthetic hoshinoamide A were in excellent agreement with the data previously reported for the natural product.

## Conclusion

In summary, we have completed the first total synthesis of hoshinoamide A. By combining the liquid and solid-phase peptide synthetic strategy, hoshinoamide A was synthesized in high efficiency. After systematic screening of the coupling reagents in liquid phase, the key intermediate tripeptide **7** was obtained in high yield. The solid-phase synthesis improves the entire efficiency of the synthetic route. This strategy could be applied to the stereoselective synthesis of hoshinoamide A and other highly methylated polypeptide analogues. Our strategy was also helpful to further study its antimalarial activity. Structure-and-activity and functional studies with the fluorescent-labeled analogs are currently under investigation in our lab.

## Experimental

**General experimental procedures.**^1^H NMR spectra were obtained using a Bruker AVANCE AV 400 spectrometer at a frequency of 400 MHz, respectively in CDCl_3_, CD_3_OD or D_2_O. Chemical shifts are reported in parts per million (ppm) and coupling constants in Hertz (Hz). The residual solvent peaks were used as internal standards. ^1^H NMR data are reported as follows: chemical shift values (ppm), multiplicity (s = singlet, d = doublet, t = triplet, q = quartet, m = multiplet), coupling constant and relative integral. ^13^C NMR spectra were obtained using a Bruker AVANCE AV 400 at 100 MHz in CDCl_3_, CD_3_OD or D_2_O. ^13^C NMR data are reported as chemical shift values (ppm).

LC–MS was performed on a Thermo Scientific MSQ instrument with the spectrometer operating in positive mode. Separations on the LC–MS system were performed on two methods using a thermo accucore C18 (2.6 µm, 100 × 2.1 mm) column. Method A: Linear gradient of 10–90% CH_3_CN/H_2_O and 0.1% TFA over 40 min were applied at a flow rate of 1.0 mL/min and detection at 220 nm. Method B: Linear gradient of 10–90–90–10% CH_3_CN/H_2_O and 0.1% TFA over 10 min (10–90 vol % MeCN over 6 min, 90–90 vol % over 3 min, 90–10 vol % MeCN over 1 min) were applied at a flow rate of 1.0 mL/min and detection at 220 nm. Preparative reverse-phase HPLC was performed by using Thermo Scientific Ultimate 3000 equipped with a Thermo Hypersil Gold (5 µm, 150 × 21.2 mm) column adpoting the following buffer systems: A: 0.1% TFA in water. B: 0.1% TFA in MeCN using a 10–90–90–10 vol % MeCN gradient (10–90 vol % MeCN over 30 min, 90–90 vol % over 10 min, 90–10 vol % MeCN over 10 min) at a flow rate of 8 mL/min.

Standard SPPS (solid-phase peptide synthesis) method:

1. General procedures for coupling on resin: The loaded resin was shaken for 2 h at room temperature with a solution of the desired Fmoc-AA-OH (4 equiv), HATU/coupling reagent (4 equiv) and DIPEA (8 equiv) in DMF (20 mL). The coupling mixture was filtered and the resin was washed with CH_2_Cl_2_ (10 mL × 5) and CH_3_OH (10 mL × 5).
2. General procedures for deprotection of Fmoc: The loaded resin was treated with a solution of 20 vol % piperidine in DMF (20 mL) for 30 min and then filtered. The resin was washed with CH_2_Cl_2_ (20 mL × 5) and CH_3_OH (20 mL × 5).
3. General procedures for cleavage the peptide from the resin: 0.5% TFA in DCM (20 mL) were added on the resin and the mixture was shaken for 2 h before filtered. The resin was washed with CH_2_Cl_2_ (20 mL × 5) and CH_3_OH (20 mL × 5).

Fmoc-Pro-OH (674 mg, 2 mmol) was then dissolved in a mixture of DCM (10 mL) and DMF (10 mL). DIPEA (1.7 mL, 10 mmol) and 2-CTC resin (1 g) were added to this mixture and the reaction was stirred at room temperature for 2 h. The resin was filtered and washed with MeOH (3 × 20 mL) and DCM (3 × 20 mL). The unreacted resin was capped with MeOH in a mixture of MeOH/DIPEA/DCM (1:2:7, 10 mL) for 3 h. The resin-bound peptide was added to a mixture of 20% piperidine in DMF (20 mL), and the mixture was shaken for 30 minutes. Then the mixture was filtered, and the resin was washed with MeOH (3 × 20 mL) and DCM (3 × 20 mL). Fmoc-*N*-Me-ᴅ-Phe-OH (1000 mg, 2.5 mmol), HCTU (1.0 g, 2.5 mmol) and DIPEA (871 µL, 5.0 mmol) in DMF were added on the resin and the reactor was shaken for 1 h at room temperature. Then the mixture was filtered, the resin was washed with MeOH (3 × 20 mL) and DCM (3 × 20 mL) to afford the resin-bound dipeptide. The resin-bound dipeptide was added to a mixture of 20% piperidine in DMF (20 mL), and the mixture was shaken for 30 minutes. Then the mixture was filtered, and the resin was washed with MeOH (3 × 20 mL) and DCM (3 × 20 mL). Fmoc-Val-OH (848 mg, 2.5 mmol), HATU (1.0 g, 2.5 mmol) and DIPEA (871 µL, 5.0 mmol) in DMF were added on the resin and the reactor was shaken for 1 h at room temperature. The resulting tripeptide was analyzed on a Thermo Scientific MSQ instrument, and few product was observed.

**Fmoc-*N*-Me-ᴅ-Phe-Pro-OBn (6).** Pro-OBn.HCl (2.41 g, 10 mmol), Fmoc-*N*-Me-ᴅ-Phe-OH (4.01 g, 10 mmol) and DIPEA (5.2 mL, 30 mmol) were dissolved in 50 mL anhydrous DCM. HATU (5.7 g, 15 mmol) was added to the solution and the mixture was stirred at room temperature for 6 h. The reaction mixture was then washed with 1.0 M HCl (20 mL), aqueous NaHCO_3_ (20 mL) and brine (20 mL). The organic phase was dried with anhydrous Na_2_SO_4_ and concentrated in vacuo. The crude residue was purified by flash column chromatography (*n*-hexanes/EA 2:1) to afford dipeptide **6** (4.9 g, 83%). ^1^H NMR (400 MHz, methanol-*d*_4_) δ 7.39–7.27 (m, 19H), 5.20–5.12 (m, 4H), 4.85 (s, 6H), 4.44 (m, 4H), 3.50 (m, 2H), 3.31–3.23 (m, 4H), 3.07 (dd, *J* = 12.8, 10.3 Hz, 2H), 2.57 (s, 6H), 2.46 (dt, *J* = 9.9, 7.0 Hz, 2H), 2.03–1.94 (m, 2H), 1.89– 1.71 (m, 4H), 1.48 (m, 2H); ^13^C NMR (101 MHz, methanol-*d*_4_) δ 171.21, 165.90, 135.71, 133.51, 129.37, 128.70, 128.24, 128.07, 127.93, 127.74, 66.74, 60.75, 59.32, 36.55, 30.88, 28.45, 23.94; HRMS–ESI (*m*/*z*): [M + H]^+^ calcd. for C_22_H_26_N_2_O_3_, 588.2671; found, 588.2673.

**Fmoc-Val-*N*-Me-ᴅ-Phe-Val-Pro-OBn (7).** To a stirred solution of dipeptide **6** (118 mg, 0.20 mmol) was added 20% Et_2_NH in CH_3_CN (5 mL) at rt for 0.5 h. The Et_2_NH and CH_3_CN were evaporated in vacuo and the residue was triturated with ether (10 mL), the crude amine was washed with ether (quintic, 10 mL each time) and dried in vacuo. The crude amine and Fmoc-Val-OH (71 mg, 0.20 mmol) were dissolved in 10 mL anhydrous DMF. The coupling reagent was added to the solution and the mixture was stirred at room temperature for 3 h. This mixture was poured onto water (10 mL) and extracted with CH_2_Cl_2_ (3 × 10 mL), then washed with 1.0 M HCl (10 mL), aqueous NaHCO_3_ (10 mL) and brine (10 mL). The organic phase was dried with anhydrous Na_2_SO_4_ and concentrated in vacuo. The crude residue was purified by flash column chromatography (*n*-hexanes/EA 2:1) to afford tripeptide **7**. ^1^H NMR (400 MHz, CDCl_3_) δ 7.75 (d, *J* = 7.7 Hz, 2H), 7.57 (t, *J* = 7.2 Hz, 2H), 7.40–7.17 (m, 14H), 5.72 (dd, *J* = 8.9, 6.5 Hz, 1H), 5.46 (d, *J* = 9.5 Hz, 1H), 5.23–5.20 (m, 1H), 5.06 (d, *J* = 12.2 Hz, 1H), 4.50–4.18 (m, 5H), 3.48–3.43 (m, 1H), 3.28 (dt, *J* = 11.3, 5.8 Hz, 2H), 3.10 (s, 1H), 2.95–2.86 (m, 3H), 2.21–2.14 (m, 2H), 1.78–1.62 (m, 5H), 1.28 (s, 2H), 0.76 (m, 3H), 0.47 (m, 3H); ^13^C NMR (101 MHz, CDCl_3_) δ 171.70, 171.60, 168.26, 156.39, 143.88, 141.30, 137.01, 129.54, 128.88, 128.69, 128.60, 128.50, 128.39, 128.31, 128.20, 127.72, 127.08, 126.65, 125.17, 125.08, 119.99, 67.04, 66.84, 59.43, 55.87, 55.59, 47.17, 46.92, 35.00, 30.72, 30.47, 28.78, 25.25, 19.82, 16.33; HRMS–ESI (*m*/*z*): [M + H]^+^ calcd. for C_42_H_45_N_3_O_6_, 688.3381; found, 688.3384. Comparison of the effects of different coupling reagents on the reaction yield is shown in Table 1.

**Fmoc-Val-*N*-Me-ᴅ-Phe-Val-Pro-OH (8).** Tripeptide **7** (2.3 g, 3.3 mmol) was dissolved in 30 mL of MeOH/HCOOH (v/v 9:1) and hydrogenized with Pd(OH)_2_ (500 mg) under H_2_ atmosphere for 10 hours to remove the benzyl groups. The reaction mixture was filtered through a pad of celite and the filtrate was concentrated in vacuo to give a brown oil which was purified by flash chromatography (*n*-hexanes/EA 2:1), affording tripeptide **8** (1.87 g, 95%) as a white foam. ^1^H NMR (400 MHz, methanol-*d*_4_) δ 7.59 (d, *J* = 8 Hz, 2H), 7.45 (d, *J* = 8 Hz, 2H), 7.21–6.96 (m, 10H), 5.52 (m, 1H), 4.81 (s, 2H), 4.20–3.98 (m, 4H), 3.29–3.10 (m, 2H), 3.04–2.91 (m, 4H), 2.80–2.67 (m, 2H), 2.62 (s, 3H), 2.01–1.83 (m, 2H), 1.53 (m, 4H), 1.13–1.09 (m, 2H), 0.52–0.45 (m, 4H); ^13^C NMR (101 MHz, methanol-*d*_4_) δ 175.49, 175.06, 174.95, 173.81, 173.14, 171.16, 170.16, 167.42, 164.85, 158.64, 158.58, 158.53, 145.38, 145.35, 145.20, 145.13, 145.09, 142.88, 142.55, 138.50, 138.41, 131.03, 130.75, 130.66, 130.27, 129.77, 129.64, 129.57, 129.42, 128.86, 128.83, 128.55, 128.28, 128.21, 127.77, 127.66, 126.35, 126.29, 126.21, 125.76, 121.02, 70.63, 68.11, 68.04, 66.85, 60.78, 60.68, 59.15, 58.19, 57.76, 57.67, 57.41, 57.06, 56.41, 56.08, 38.96, 37.58, 37.01, 36.60, 35.79, 35.61, 33.11, 32.20, 31.89, 31.73, 31.53, 31.40, 30.88, 30.83, 30.66, 30.53, 30.39, 29.98, 29.64, 28.18, 26.97, 26.10, 23.80, 23.34, 19.88, 19.48, 18.17, 18.07, 17.64, 14.58; HRMS–ESI (*m*/*z*): [M + H]^+^ calcd. for C_35_H_39_N_3_O_6_, 598.2912; found, 598.2915.

### Solid-phase synthesis of hoshinoamide A from tripeptide **8**

Tripeptide **8** (1.20 g, 2 mmol) was dissolved in a mixture of DCM (10 mL) and DMF (10 mL). DIPEA (1.7 mL, 10 mmol) and 2-CTC resin (1 g) were added to this mixture and the reaction was stirred at room temperature for 3 h. The resin was filtered and washed with MeOH (3 × 20 mL) and DCM (3 × 20 mL). Tripeptide **8** was recovered (1.6 mmol). The unreacted resin was capped with MeOH in a mixture of MeOH/DIPEA/DCM (1:2:7, 10 mL) for 5 h. The Fmoc protecting group was removed following the general procedure and the remaining amino acids were successively coupled using the standard SPPS method. 0.5% TFA in DCM (20 mL) was added on the resin and the mixture was shaken for 2 h to cleave the peptide from the resin. The mixture was filtered and the filtrate was concentrated in vacuo to give a white foam. The peptide was re-dissolved in a mixture of TFA/Et_3_SiH/H_2_O (10 mL, 50:50:50 v/v/v). The reaction mixture was stirred for 3 h, and then concentrated in vacuo. The crude peptide was precipitated using cold Et_2_O and centrifuged at 7000 rpm to give a white solid. This solid was further purified by RP-HPLC through protocols described in the general method section. Fractions were collected, concentrated and lyophilized to give nanopeptide **10** as a white solid. Some nanopeptide **10** (23 mg, 0.02 mmol) was dissolved in dry DMF (5 mL). The remaining nanopeptide **10** was recovered. K_2_CO_3_ (3.1 mg, 0.022 mmol) and MeI (3.13 mg, 0.022 mmol) was added to this solution. The reaction mixture was stirred for 3 h. This mixture was poured onto water (5 mL) and extracted with CH_2_Cl_2_ (3 × 5 mL). then washed with 1.0 M HCl (10 mL), aqueous NaHCO_3_ (10 mL) and brine (10 mL). The organic phase was dried with anhydrous Na_2_SO_4_ and concentrated in vacuo to give a brown oil. This oil was further purified by RP-HPLC using protocols described in the general method section. Fractions were collected, concentrated and lyophilized to give hoshinoamide A as a white solid (10 mg, 2% yield). The ^1^H NMR and ^13^C NMR spectra of the synthetic product were fully consistent with the data of isolated samples reported in the literature [11]. See Supporting Information File 1, Tables S2 and S3 for details. ^1^H NMR (400 MHz, methanol-*d*_4_) δ 7.30–7.11 (m, 6H), 7.02–6.94 (m, 2H), 6.72–6.65 (m, 2H), 5.73 (dd, *J* = 9.2, 6.4 Hz, 1H), 4.82–4.73 (m, 1H), 4.64–4.53 (m, 2H), 4.43–4.25 (m, 2H), 3.69 (s, 2H), 3.50 (dt, *J* = 11.2, 6.0 Hz, 1H), 3.41–3.31 (m, 1H), 3.20–3.06 (m, 9H), 2.99–2.92 (m, 3H), 2.91–2.86 (m, 1H), 2.52 (t, *J* = 7.6 Hz, 2H), 2.47–2.38 (m, 2H), 2.29–2.22 (m, 3H), 2.18 (q, *J* = 7.7 Hz, 3H), 2.05–1.99 (m, 1H), 1.99–1.71 (m, 9H), 1.68–1.46 (m, 6H), 1.46–1.34 (m, 4H), 1.11–0.97 (m, 3H), 0.97–0.81 (m, 16H), 0.68–0.55 (m, 6H). ^13^C NMR (101 MHz, methanol-*d*_4_) δ 177.43, 176.52, 176.00, 174.55, 173.86, 173.15, 173.09, 172.87, 171.89, 170.47, 156.60, 138.40, 133.75, 130.69, 130.40, 129.45, 127.70, 116.18, 64.21, 60.84, 57.23, 55.69, 55.64, 55.52, 54.32, 52.69, 40.23, 38.43, 37.82, 36.62, 35.76, 35.51, 34.59, 32.62, 31.80, 31.59, 31.56, 30.22, 29.98, 29.20, 27.72, 26.21, 26.17, 26.06, 25.95, 25.90, 25.11, 23.80, 22.17, 20.39, 19.90, 19.49, 18.13, 16.50, 11.60; HRMS–ESI (*m*/*z*): [M + H]^+^ calcd. for C_61_H_95_N_9_O_12_, 1146.7173; found, 1146.7173.

## Supporting Information

File 1 NMR (^1^H NMR and ^13^C NMR) spectra of compounds **2**−**8**, and comparison of the spectral data of natural and synthetic hoshinoamide A.
