# Supplementary material for: First total synthesis of hoshinoamide A
Source: Beilstein J Org Chem. 2021 Dec 15;17:2924–31. doi: 10.3762/bjoc.17.201 (PMC8685559; doi:10.3762/bjoc.17.201)
Supplement: File 1 — NMR (1H NMR and 13C NMR) spectra of compounds 2−8, and comparison of the spectral data of natural and synthetic hoshinoamide A. [file Beilstein_J_Org_Chem-17-2924-s001.pdf]

BEILSTEIN JOURNAL OF ORGANIC CHEMISTRY

Supporting Information

for

First total synthesis of hoshinoamide A

Haipin Zhou, Zihan Rui, Yiming Yang, Shengtao Xu, Yutian Shao and Long Liu

Beilstein J. Org. Chem. **2021,** *17,* 2924–2931. doi:10.3762/bjoc.17.201

NMR (^{1}H NMR and ^{13}C NMR) spectra of compounds 2−8, and comparison of the spectral data of natural and synthetic hoshinoamide A

License and Terms: This is a supporting information file under the terms of the Creative Commons Attribution License (https://creativecommons.org/licenses/by/4.0). Please note that the reuse, redistribution and reproduction in particular requires that the author(s) and source are credited and that individual graphics may be subject to special legal provisions.
The license is subject to the *Beilstein Journal of Organic Chemistry* terms and conditions: (https://www.beilstein-journals.org/bjoc/terms)

Table of contents

1. Experimental and analytical data S2

1.1 Synthesis of Fmoc-*N*-Me-D-Phe/Val-OH and Fmoc-Aha-OH.....................S2

1.2 Solid phase synthesis of intermediate A...S5

1.3 Synthesis of tripeptide intermediate S6

1.4 Synthesis of hoshinoamide A S7

2. References..S12

3. ^{1}H NMR and ^{13}C NMR Spectra...S13

4. ^{1}H NMR and ^{13}C NMR spectra, high resolution mass spectra, and analytical HPLC spectra of hoshinoamide A S20

1. Experimental and analytical data

1.1 Synthesis of Fmoc-*N*-Me-D-Phe/Val-OH and Fmoc-Aha-OH

Fmoc-*N*-Me-D-Phe-OH and Fmoc-*N*-Me-D-Val-OH were synthesized using a modified procedure[1] reported by Boc-D-Phe-OH [2] and Boc-D-Val-OH [2]. Fmoc-Aha-OH was synthesized using a modified procedure reported by aminocaproic acid [3]. All data for known compounds are consistent with those reported in literature.

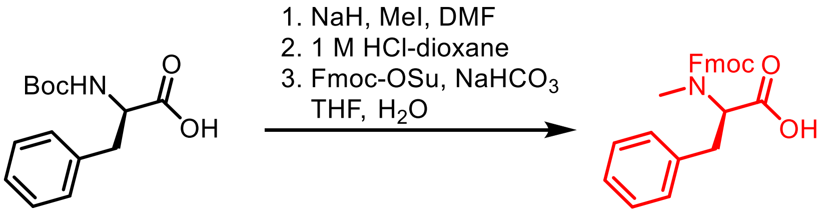

Scheme S1: Synthesis of Fmoc-*N*-Me-D-Phe.

Boc-D-Phe-OH (5.00 g, 18.9 mmol) and MeI (1.8 mL, 28.4 mmol)were dissolved in DMF (50 mL). The reaction mixture was stirred at 0 °C for 45 min, then NaH (2.27 g, 56.7 mmol, 60%) was slowly added to the reaction mixture. The reaction mixture was stirred at room temperature for an additional 3 h, then quenched with water (150 mL) and extracted with ethyl acetate (3 × 50 mL). The combined organic phases were washed with water (100 mL) and brine (100 mL). The organic phase was dried over Na_2SO_4, filtered and concentrated to afford Boc-*N*-Me-D-Phe-OH which was used without purification.

Boc-*N*-Me-D-Phe-OH was dissolved in 10% HCl-dioxanne (50 mL). The mixture was stirred at room temperature for 4 h, then concentrated to give a brown oil. The resulting crude oil was azeotroped with toluene (3 × 10 mL) and concentrated in vacuo to remove any residual HCl. The concentrated crude material was then dissolved in a mixture of THF (25 mL), H_2O (25 mL) and $NaHCO_3$ (3.2 g, 37.8 mmol), Fmoc-OSu (9.5 g, 28.4 mmol) was added to this mixture and the reaction mixture was stirred at room temperature for 8 h. Then added with water (150 mL) and extracted with ethyl acetate (3 × 50 mL). The combined organic phases were washed with water (100 mL) and brine (100 mL). The mixture was concentrated to give a

crude white foam which was purified by flash chromatography (*n*-hexanes/EA = 2:1) to afford Fmoc-*N*-Me-D-Phe-OH (5.6 g, 14.0 mmol, 74%) as a white foam.**^{1}H NMR** (400 MHz, DMSO-d_6) δ 12.97 (s, 1H), 7.85 (d, *J* = 7.4 Hz, 2H), 7.52 (m, 2H), 7.40 (q, *J* = 7.3 Hz, 2H), 7.33-7.05 (m, 7H), 4.79 (m, 4.7 Hz, 1H), 4.24 (m, 3H), 3.33-2.82 (m, 2H), 2.72 (d, *J* = 6.5 Hz, 3H). **^{13}C NMR** (101 MHz, DMSO-d_6) δ 172.56, 172.41, 156.23, 156.03, 144.35, 144.25, 144.13, 141.23, 141.20, 138.38, 138.21, 129.24, 129.21, 128.74, 128.10, 127.57, 126.82, 125.52, 125.42, 125.36, 120.54, 67.31, 60.70, 60.16, 47.18, 47.03, 34.85, 34.56, 32.19, 31.79. **HRMS:** (+ESI) Calc. for $C_{25}H_{23}NO_4$: 402.1700 $[M+H]^+$, Found: 402.1698 $[M+H]^+$.

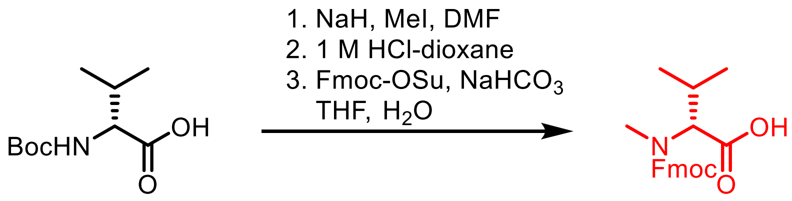

Scheme S2: Synthesis of Fmoc-*N*-Me-D-Val-OH.

Boc-D-Val-OH (5.0 g, 23.0 mmol) and MeI (2.2 mL, 28.4 mmol)were dissolved in DMF (50 mL). The reaction mixture was stirred at 0 °C for 45 min, then NaH (2.7 g, 68.0 mmol, 60%) was slowly added to the reaction mixture. The reaction mixture was stirred at room temperature for an additional 5 h, then quenched with water (150 mL) and extracted with ethyl acetate (3 × 50 mL). The combined organic phases were washed with water (100 mL) and brine (100 mL). The organic phase was dried over Na_2SO_4, filtered and concentrated to afford Boc-*N*-Me-D-Val-OH which was used without purification.

Boc-*N*-Me-D-Val-OH was dissolved in 10% HCl-dioxanne (50 mL). The mixture was stirred at room temperature for 3 h, then concentrated to give a brown oil. The resulting crude oil was azeotroped with toluene (3 × 10 mL) and concentrated in vacuo to remove any residual HCl. The concentrated crude material was then dissolved in a mixture of THF (25 mL), H_2O (25 mL) and $NaHCO_3$ (3.8 g, 45.4 mmol), Fmoc-OSu (11.4 g, 34.1 mmol) was added to this mixture and the reaction mixture was stirred at room temperature for 8 h. Then added with water (150 mL) and

extracted with ethyl acetate (3 × 50 mL). The combined organic phases were washed with water (100 mL) and brine (100 mL). The mixture was concentrated to give a crude white foam which was purified by flash chromatography (DCM/MeOH = 100:1) to afford **Fmoc-*N*-Me-D-Val-OH** (6.6 g, 18.6 mmol, 81%) as a white foam.**^{1}H NMR** (400 MHz, DMSO-d_6) δ 12.78 (s, 1H), 7.85 (d, *J* = 7.1 Hz, 2H), 7.71-7.58 (m, 2H), 7.38 (t, *J* = 7.1 Hz, 2H), 7.31 (t, *J* = 7.1 Hz, 2H), 4.39 (m, 2H), 4.25 (m, 2H), 2.76 (s, 3H), 2.05 (m, 1H), 0.99-0.56 (m, 6H). **^{13}C NMR** (101 MHz, DMSO-d_6) δ 172.50, 172.33, 156.61, 156.00, 144.39, 144.25, 141.37, 128.13, 127.58, 125.49, 125.44, 120.56, 67.34, 64.33, 47.33, 47.26, 30.66, 27.49, 27.37, 20.22, 20.15, 19.32, 19.02. **HRMS:** (+ESI) Calc. for $C_{21}H_{23}NO_4$: 354.1700 $[M+H]^+$, Found: 354.1696 $[M+H]^+$.

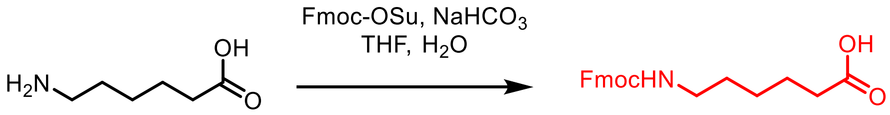

Scheme S3: Synthesis of Fmoc-Aha-OH

Aminocaproic acid (2.0g, 15.2 mmol) dissolved in a mixture of THF (25 mL), H_2O (25 mL) and $NaHCO_3$ (2.5 g, 30.4 mmol), Fmoc-OSu (10.2 g, 30.4 mmol) was added to this mixture and the reaction mixture was stirred at room temperature for 7 h. Then added with water (150 mL) and extracted with ethyl acetate (3 × 50 mL). The combined organic phases were washed with water (100 mL) and brine (100 mL). The mixture was concentrated to give a crude white foam which was purified by flash chromatography (DCM:MeOH = 50:1) to afford Fmoc-Aha-OH (4.8 g, 13.7 mmol, 90%) as a white foam. **^{1}H NMR** (400 MHz, $CDCl_3$) δ 10.70 (s, 1H), 7.71 (d, *J* = 7.5 Hz, 2H), 7.55 (d, *J* = 6.9 Hz, 2H), 7.31 (m, 4H), 4.97 (s, 1H), 4.53-4.30 (m, 2H), 4.24 -4.09 (m, 1H), 3.13 (d, *J* = 6.0 Hz, 1H), 3.00 (s, 1H), 2.29 (t, *J* = 7.2 Hz, 2H), 1.67-1.51 (m, 2H), 1.50-1.40 (m, 1H), 1.39-1.15 (m, 3H). **^{13}C NMR** (101 MHz, $CDCl_3$) δ 179.02, 157.88, 156.62, 143.98, 141.31, 127.68, 127.05, 125.05, 124.87, 119.97, 67.18, 66.57, 47.27, 41.35, 40.80, 34.06, 29.53, 26.13, 24.34. **HRMS:** (+ESI) Calc. for $C_{21}H_{23}NO_4$: 354.1700 $[M+H]^+$, Found: 354.1696 $[M+H]^+$.

1.2 Solid phase synthesis of tripeptide 3

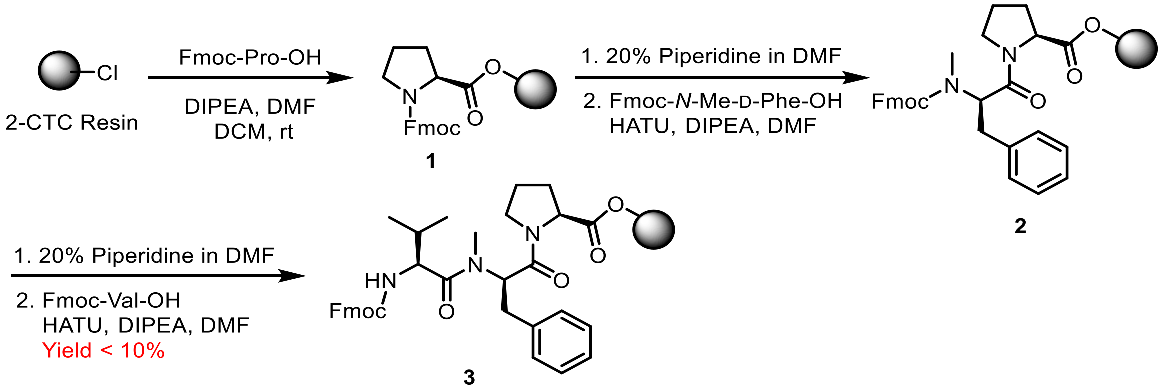

Scheme S4: Solid phase synthesis of tripeptide 3

Fmoc-Pro-OH (674 mg, 2 mmol) was then dissolved in a mixture of DCM (10 mL) and DMF (10 mL). DIPEA (1.7 mL, 10 mmol), 2-CTC resin (1 g) were added to this mixture and the reaction was stirred at room temperature was for 2h. The resin was filtered and washed with MeOH (3 × 20 mL), DCM (3 × 20 mL). The unreacted resin was capped with MeOH in a mixture of MeOH:DIPEA:DCM (1:2:7, 10 mL) for 3h. The resin-bound peptide was added to a mixture of 20% piperidine in DMF (20 mL), and the mixture was shaken to for 30 minutes. Then the mixture was filtered, the resin was washed with MeOH (3 × 20 mL) and DCM (3 × 20 mL). Fmoc-*N*-Me-D-Phe-OH (1000 mg, 2.5 mmol), HATU (950 mg, 2.5 mmol) and DIPEA (871 µL, 5.0 mmol) in DMF were added on the resin and the reactor was shaken for 1h at room temperature. Then the mixture was filtered, the resin was washed with MeOH (3 × 20 mL) and DCM (3 × 20 mL) to afford the resin-bound dipeptide. The resin-bound dipeptide was added to a mixture of 20% piperidine in DMF (20 mL), and the mixture was shaken to for 30 minutes. Then the mixture was filtered, the resin was washed with MeOH (3 × 20 mL) and DCM (3 × 20 mL). Fmoc-Val-OH (848 mg, 2.5 mmol), HATU (950 mg, 2.5 mmol) and DIPEA (871 µL, 5.0 mmol) in DMF were added on the resin and the reactor was shaken for 1h at room temperature. The resulting tripeptide **3** was analysed on a Thermo Scientific MSQ instrument, and few product was observed.

1.3 Synthesis of tripeptide 7

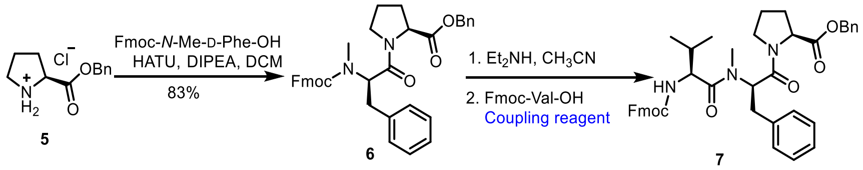

Scheme S5: synthesis of tripeptide 7.

Pro-OBn.HCl (2.41 g, 10 mmol), Fmoc-*N*-Me-D-Phe-OH (4.01 g, 10 mmol) and DIPEA (5.2 mL, 30 mmol) was dissolved in 50 mL anhydrous DCM. HATU (5.7 g, 15 mmol) was added to the solution and the mixture was stirred at room temperature for 6 h. The reaction mixture was then washed by 1.0 M HCl (20 mL), aqueous $NaHCO_3$ (20 mL) and brine (20 mL). The organic phase was dried with anhydrous Na_2SO_4 and concentrated in vacuo. The crude residue was purified by flash column chromatography (*n*-hexanes/EA = 2:1) to afford dipeptide **6** (4.9 g, 83%). **^{1}H NMR** (400 MHz, Methanol-d_4) δ 7.39-7.27 (m, 19H), 5.20- 5.12 (m, 4H), 4.85 (s, 6H), 4.44 (m, 4H), 3.50 (m, 2H), 3.31-3.23 (m, 4H), 3.07 (dd, *J* = 12.8, 10.3 Hz, 2H), 2.57 (s, 6H), 2.46 (dt, *J* = 9.9, 7.0 Hz, 2H), 2.03-1.94 (m, 2H), 1.89-1.71 (m, 4H), 1.48 (m, 2H). ^{13}C NMR (101 MHz, Methanol-d_4) δ 171.21, 165.90, 135.71, 133.51, 129.37, 128.70, 128.24, 128.07, 127.93, 127.74, 66.74, 60.75, 59.32, 36.55, 30.88, 28.45, 23.94. HRMS: (+ESI) Calc. for $C_{22}H_{26}N_2O_3$: 588.2671[M+H]$^+$, Found:588.2673[M+H]$^+$.

To a stirred solution of dipeptide **6** (118 mg, 0.20 mmol) was added 20% Et_2NH in CH_3CN (5 mL) at rt for 0.5 h. The Et_2NH and CH_3CN are evaporated in vacuo and the residue is triturated with ether (10 mL), the crude amine washed with ether (quintic, 10 mL each time) and dried in vacuo. To a stirred solution of crude amine and Fmoc-Val-OH (71 mg, 0.20 mmol) was dissolved in 10 mL anhydrous DMF. Coupling reagents was added to the solution and the mixture was stirred at room temperature for 3 h. This mixture poured onto water (10 mL) and extracted with CH_2Cl_2 (3 × 10 mL). Then washed by 1.0 M HCl (10 mL), aqueous $NaHCO_3$ (10 mL) and brine (10 mL). The organic phase was dried with anhydrous Na_2SO_4 and concentrated in vacuo. The crude residue was purified by flash column

chromatography (*n*-hexanes/EA = 2:1) to afford tripeptide **7**. ^{1}H NMR (400 MHz, $CDCl_3$) δ 7.75 (d, *J* = 7.7 Hz, 2H), 7.57 (t, *J* = 7.2 Hz, 2H), 7.40-7.17 (m, 14H), 5.72 (dd, *J* = 8.9, 6.5 Hz, 1H), 5.46 (d, *J* = 9.5 Hz, 1H), 5.23-5.20 (m, 1H), 5.06 (d, *J* = 12.2 Hz, 1H), 4.50-4.18 (m, 5H), 3.48-3.43 (m, 1H), 3.28 (dt, *J* = 11.3, 5.8 Hz, 2H), 3.10 (s, 1H), 2.95-2.86 (m, 3H), 2.21-2.14 (m, 2H), 1.78-1.62 (m, 5H), 1.28 (s, 2H), 0.76 (m, 3H), 0.47 (m, 6.7 Hz, 3H). ^{13}C NMR (101 MHz, $CDCl_3$) δ 171.70, 171.60, 168.26, 156.39, 143.88, 141.30, 137.01, 129.54, 128.88, 128.69, 128.60, 128.50, 128.39, 128.31, 128.20, 127.72, 127.08, 126.65, 125.17, 125.08, 119.99, 67.04, 66.84, 59.43, 55.87, 55.59, 47.17, 46.92, 35.00, 30.72, 30.47, 28.78, 25.25, 19.82, 16.33. HRMS**:** (+ESI) Calc. for $C_{42}H_{45}N_3O_6$: 688.3381 $[M+H]^+$, Found:688.3384 $[M+H]^+$. Comparison of the effects of different coupling reagents on the reaction yield (Table S1).

1.4 Synthesis of hoshinoamide A

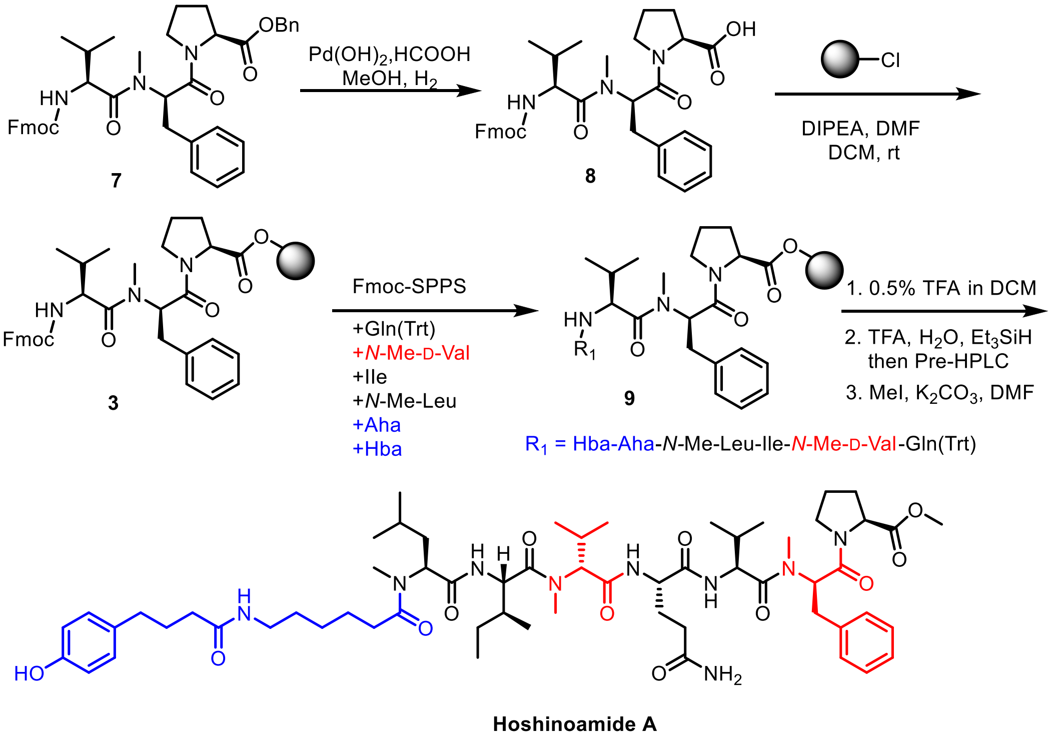

Scheme S6: synthesis of hoshinoamide A.

Tripeptide **7** (2.3 g, 3.3 mmol) was was dissolved in 30 mL of MeOH/HCOOH(v/v = 9:1) and hydrogenized with $Pd(OH)_2$ (500 mg) under H_2 for 10 hours to remove the Bn groups. The reaction mixture was filtered through a pad of celite and the filtrate was concentrated in vacuo to a give brown oil which was

purified by flash chromatography (*n*-hexanes:EA = 2:1), affording tripeptide **8** (1.87 g, 95%) as a white foam. ^{1}H NMR (400 MHz, Methanol-d_4) δ 7.59 (d, *J* = 8 Hz, 2H), 7.45 (d, *J* = 8 Hz, 2H), 7.21-6.96 (m, 10H), 5.52 (m, 1H), 4.81 (s, 2H), 4.20-3.98 (m, 4H), 3.29-3.10 (m, 2H), 3.04–2.91 (m, 4H), 2.80-2.67 (m, 2H), 2.62 (s, 3H), 2.01-1.83 (m, 2H), 1.53 (m, 4H), 1.13-1.09 (m, 2H), 0.52-0.45 (m, 4H). ^{13}C NMR (101 MHz, Methanol-d_4) δ 175.49, 175.06, 174.95, 173.81, 173.14, 171.16, 170.16, 167.42, 164.85, 158.64, 158.58, 158.53, 145.38, 145.35, 145.20, 145.13, 145.09, 142.88, 142.55, 138.50, 138.41, 131.03, 130.75, 130.66, 130.27, 129.77, 129.64, 129.57, 129.42, 128.86, 128.83, 128.55, 128.28, 128.21, 127.77, 127.66, 126.35, 126.29, 126.21, 125.76, 121.02, 70.63, 68.11, 68.04, 66.85, 60.78, 60.68, 59.15, 58.19, 57.76, 57.67, 57.41, 57.06, 56.41, 56.08, 38.96, 37.58, 37.01, 36.60, 35.79, 35.61, 33.11, 32.20, 31.89, 31.73, 31.53, 31.40, 30.88, 30.83, 30.66, 30.53, 30.39, 29.98, 29.64, 28.18, 26.97, 26.10, 23.80, 23.34, 19.88, 19.48, 18.17, 18.07, 17.64, 14.58. **HRMS:** (+ESI) Calc. for $C_{35}H_{39}N_3O_6$: 598.2912 $[M+H]^+$, Found: 598.2915$[M+H]^+$.

Tripeptide **8** (1.20 g, 2 mmol) was then dissolved in a mixture of DCM (10 mL) and DMF (10 mL). DIPEA (1.7 mL, 10 mmol), 2-CTC resin (1 g) were added to this mixture and the reaction was stirred at room temperature was for 3h. The resin was filtered and washed with MeOH (3 × 20 mL), DCM (3 × 20 mL). The unreacted resin was capped with MeOH in a mixture of MeOH:DIPEA:DCM (1:2:7, 10 mL) for 5 h. Fmoc protecting group was removed following the general procedure and the remain amino acids were successively coupled using the standard SPPS method. (Ile_6 and Aha_8 are coupled with coupling reagengt. Gln^4, *N*-Me-D-Val^5, *N*-Me-L-Leu^7 and Hba^9 are coupled with HATU and DIPEA.) 0.5% TFA in DCM (20 mL) were added on the resin and the mixture was shaken for 2h to cleavage the peptide from the resin. The mixture was filtered and the filtrate was concentrated *in vacuo* to give a white foam. The peptide was re-dissolved in a mixture of TFA:Et_3SiH:H_2O (10 mL, 50/50/50 v/v/v). The reaction mixture was stirred for 3 h, and then concentrated *in vacuo*. The crude peptide was precipitated using cold Et_2O and centrifuged at 7000 rpm to give a white solid. This solid was further purified by RP-HPLC using protocols described in the general method. Fractions were collected, concentrated and

lyophilized to give nanopeptide 10 as a white solid. Nanopetide 10 was dissolved in dry DMF (5 mL). K_2CO_3 (3.1 mg, 0.022 mmol) and MeI (3.13 mg, 0.022 mmol) was added to this solution. The reaction mixture was stirred for 3 h. This mixture poured onto water (5 mL) and extracted with CH_2Cl_2 (3 × 5 mL). Then washed by 1.0 M HCl (10 mL), aqueous $NaHCO_3$ (10 mL) and brine (10 mL). The organic phase was dried with anhydrous Na_2SO_4 and concentrated in vacuoto give brown oil . This oil was further purified by RP-HPLC using protocols described in the general method. Fractions were collected, concentrated and lyophilized to give **Hoshinoamide A** as a white solid.(10 mg, 2% yield). HRMS: (+ESI) Calc. for $C_{61}H_{95}N_9O_{12}$: 1146.7173 $[M+H]^+$, Found: 1146.7173 $[M+H]^+$; The ^1H NMR and ^{13}C NMR spectra of synthetic product were fully consistent with the data of isolated samples reported in the literature [4]. See table S2 and table S3 for details. **^{1}H NMR** (400 MHz, Methanol-d_4) δ 7.30 - 7.11 (m, 6H), 7.02 - 6.94 (m, 2H), 6.72 - 6.65 (m, 2H), 5.73 (dd, J = 9.2, 6.4 Hz, 1H), 4.82 - 4.73 (m, 1H), 4.64 - 4.53 (m, 2H), 4.43 - 4.25 (m, 2H), 3.69 (s, 2H), 3.50 (dt, J = 11.2, 6.0 Hz, 1H), 3.41 – 3.31 (m, 1H), 3.20 – 3.06 (m, 9H), 2.99 – 2.92 (m, 3H), 2.91 – 2.86 (m, 1H), 2.52 (t, J = 7.6 Hz, 2H), 2.47 – 2.38 (m, 2H), 2.29 – 2.22 (m, 3H), 2.18 (q, J = 7.7 Hz, 3H), 2.05 – 1.99 (m, 1H), 1.99 – 1.71 (m, 9H), 1.68 – 1.46 (m, 6H), 1.46 – 1.34 (m, 4H), 1.11 – 0.97 (m, 3H), 0.97 – 0.81 (m, 16H), 0.68 – 0.55 (m, 6H). **^{13}C NMR** (101 MHz, Methanol-d_4) δ 177.43, 176.52, 176.00, 174.55, 173.86, 173.15, 173.09, 172.87, 171.89, 170.47, 156.60, 138.40, 133.75, 130.69, 130.40, 129.45, 127.70, 116.18, 64.21, 60.84, 57.23, 55.69, 55.64, 55.52, 54.32, 52.69, 40.23, 38.43, 37.82, 36.62, 35.76, 35.51, 34.59, 32.62, 31.80, 31.59, 31.56, 30.22, 29.98, 29.20, 27.72, 26.21, 26.17, 26.06, 25.95, 25.90, 25.11, 23.80, 22.17, 20.39, 19.90, 19.49, 18.13, 16.50, 11.60. **HRMS**: (+ESI) Calc. for $C_{61}H_{95}N_9O_{12}$: 1146.7173 $[M+H]^+$, Found: 1146.7173 $[M+H]^+$;

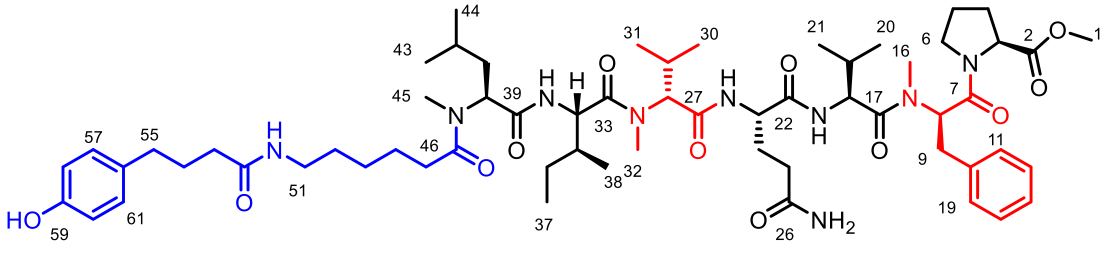

Table S1. ^{1}H and ^{13}C NMR Spectroscopic Data of Naturally Occurring and Synthetic Hoshinoamide A

Postition	natural product		synthetic product	
	δH (CD_3OD, 400 MHz)	δC (CD_3OD, 100 MHz)	δH (CD_3OD, 400 MHz)	δC (CD_3OD, 100 MHz)
1	3.7	52.7	3.69	52.7
2		173.8		173.9
3	4.38	60.8	4.38	60.8
4a	2.23	29.7	2.24	
4b	1.86		1.85	
5a	1.97	26.2	1.96	26.2
5b	1.87		1.87	
6a	3.5	48.2	3.50	
6b	3.34		3.35	
7		170.5		170.5
8	5.74	57.2	5.74	57.2
9a	3.17	35.7	3.16	35.7
9b	2.9		2.89	
10		138.4		138.4
11	7.24	130.7	7.24	130.7
12	7.25	129.4	7.25	129.5
13	7.16	127.7	7.16	127.7
16	3.09	31.5	3.08	31.6
17		173.14		173.15
18	4.57	55.6	4.57	55.6
19	1.76	31.5	1.77	31.6
20	0.65	18.1	0.64	18.1
21	0.6	19.9	0.59	19.9
22		173.06		173.09
23	4.3	54.3	4.30	54.3
24a	2.02	26	2.01	26
24b	1.93		1.93	
25a	2.25	29.9	2.25	30.0
25b	1.87		1.87	
26		177.4		177.4

27		171.9		171.9
28	4.61	64.1	4.61	64.2
29	2.28	27.7	2.27	27.7
30	0.98	20.3	0.98	20.4
31	0.85	19.5	0.85	19.5
32	3.09	32.6	3.08	32.6
33		174.5		174.6
34	4.78	55.5	4.78	55.5
35	1.84	38.4	1.83	38.4
36a	1.46	25.1	1.46	25.1
36b	1.07		1.07	
37	0.86	11.6	0.85	11.6
38	0.93	16.5	0.93	16.5
39		172.9		172.9
40	5.52	55.6	5.52	55.7
41a	1.75	37.8	1.75	37.8
41b	1.59		1.59	
42	1.4	26.7	1.40	
43	0.94	23.8	0.94	23.8
44	0.89	22.1	0.89	22.2
45	2.97	31.8	2.96	31.8
46		176.5		176.5
47	2.43	34.6	2.42	34.6
48	1.64	25.9	1.63	25.9
49	1.39	27.7	1.39	
50	1.53	30.2	1.52	30.2
51	3.17	40.2	3.16	40.2
52		176		176
53	2.16	36.6	2.17	36.6
54	1.86	29.2	1.86	29.2
55	2.52	35.5	2.52	35.5
56		133.7		133.8
57/61	6.99	130.4	6.99	130.4
58/60	6.7	116.1	6.69	116.2
59		156.6		156.6

2. References

[1] Qi, N.; Allu, S.; Wang, Z.; Liu, Q.; Guo, J.; He, Y. Asymmetric Total Syntheses of Aetheramides and Their Stereoisomers: Stereochemical Assignment of Aetheramides. *Org. Lett*. **2016**, *18*, 4718–4721.

[2] Buba, A.; Koch, S.; Kunz, H.; Loewe, H. Fluorenylmethoxy-carbonyl-N-methylamino Acids Synthesized in a Flow Tube-in-Tube Reactor with a Liquid-Liquid Semipermeable Membrane. *Eur. J. Org. Chem.* **2013**, 21, 4509-4513

[3] Ellison, J.; Raines, R. A pendant peptide endows a sunscreen with water-resistance. *Org. Biomol. Chem.* 2018, 16, 7139-7142

[4] Iwasaki, A.; Tadenuma, T.; Sumimoto, S.; Shiota, I.; Matsubara, T.; Saito-Nakano, Y.; Nozaki, T.; Sato, T.; Suenaga, K. Hoshinoamides A and B, Acyclic Lipopeptides from the Marine Cyanobacterium Caldora penicillata. *J. Nat. Prod*. **2018**, 81, 2545−2552.

3、^{1}H-NMR and ^{13}C-NMR Spectra

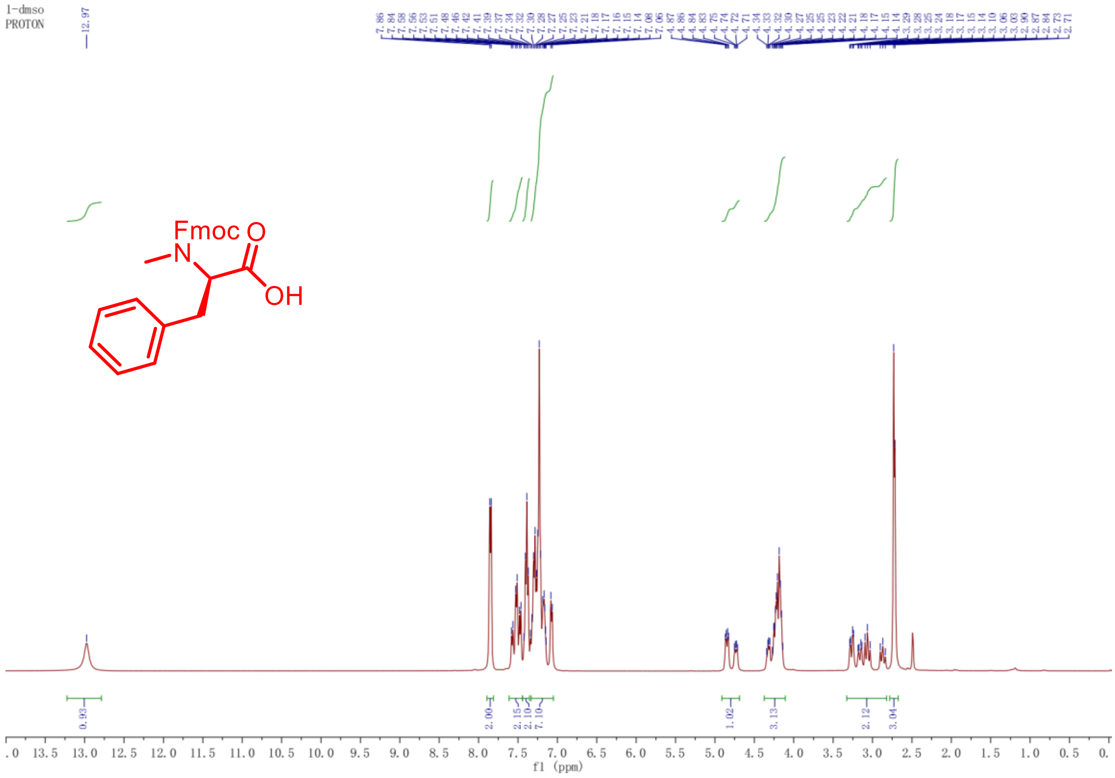

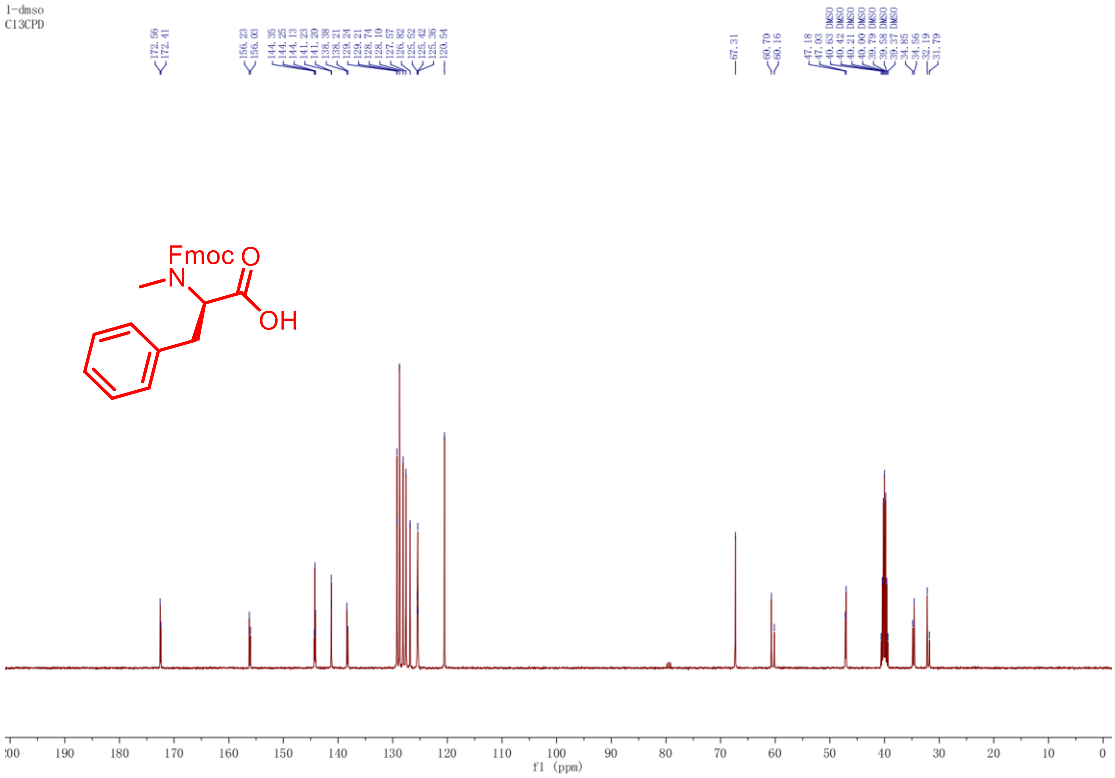

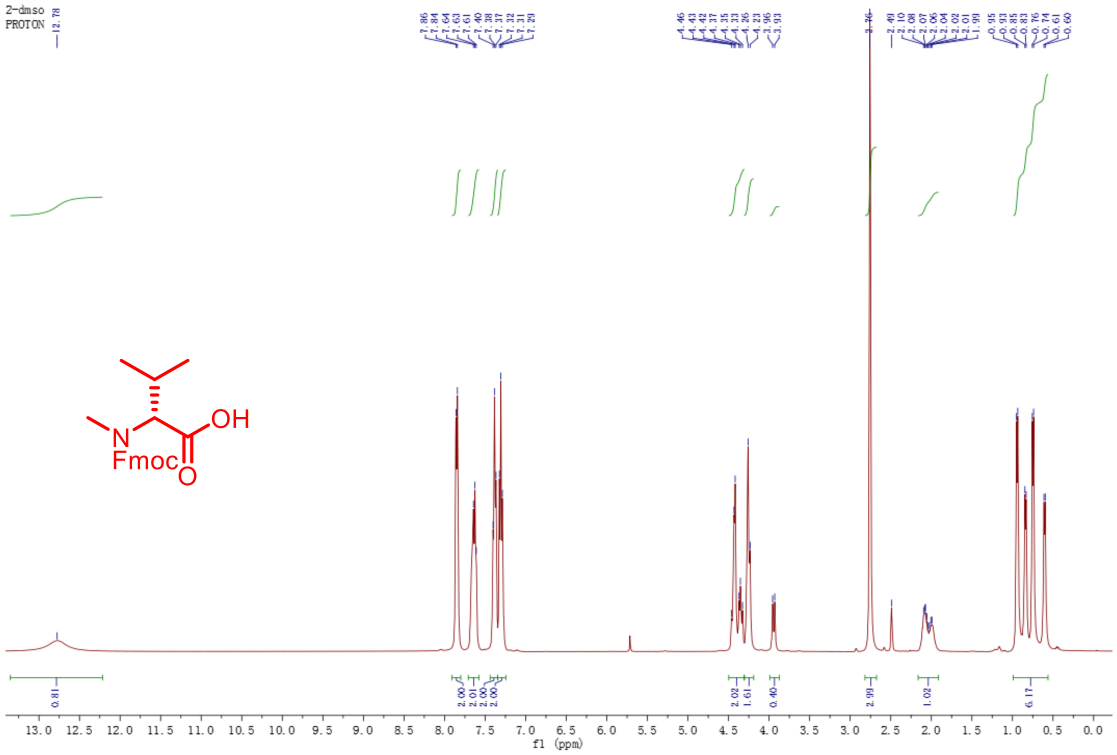
2-dmso
PROTON
OH
N
Fmoc
O
f1 (ppm)

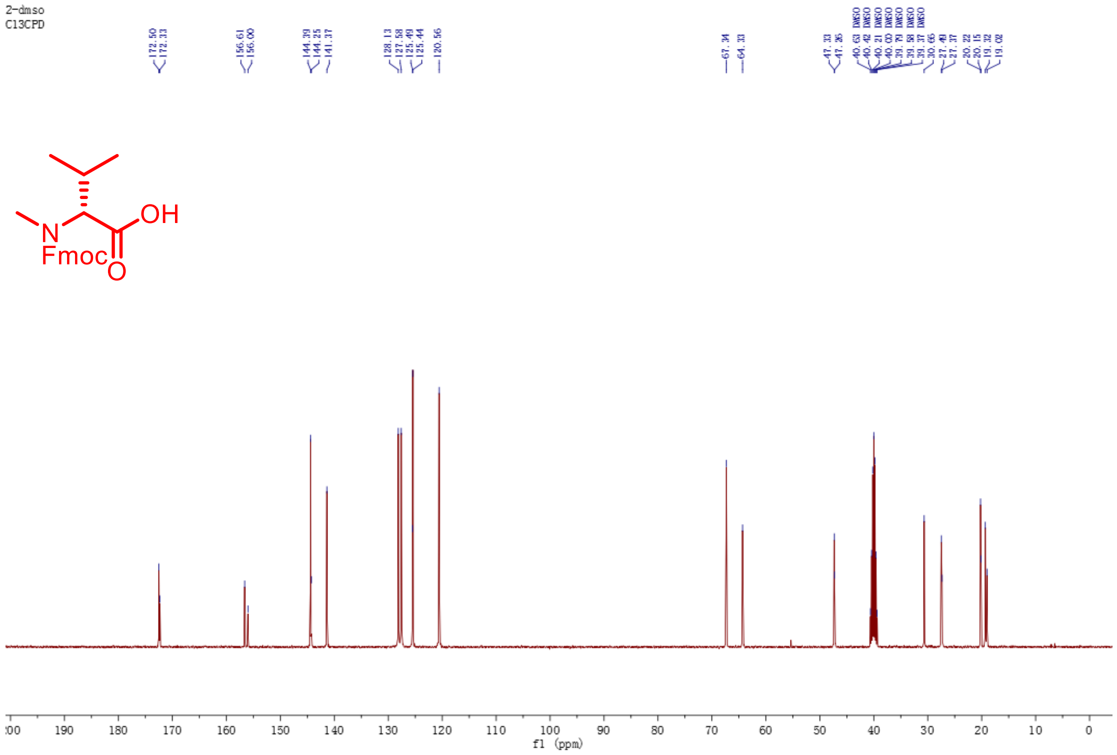
2-dmso
C13CPD
OH
N
Fmoc
O
f1 (ppm)

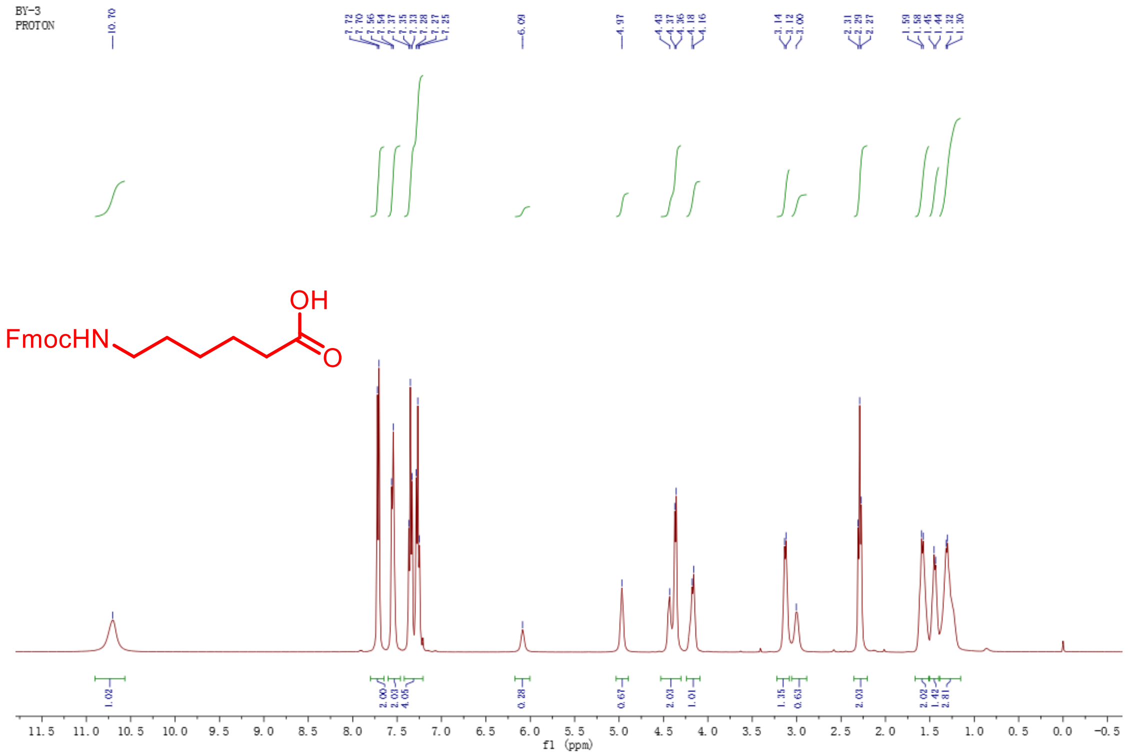
BY-3
PROTON
FmocHN
OH
O
f1 (ppm)

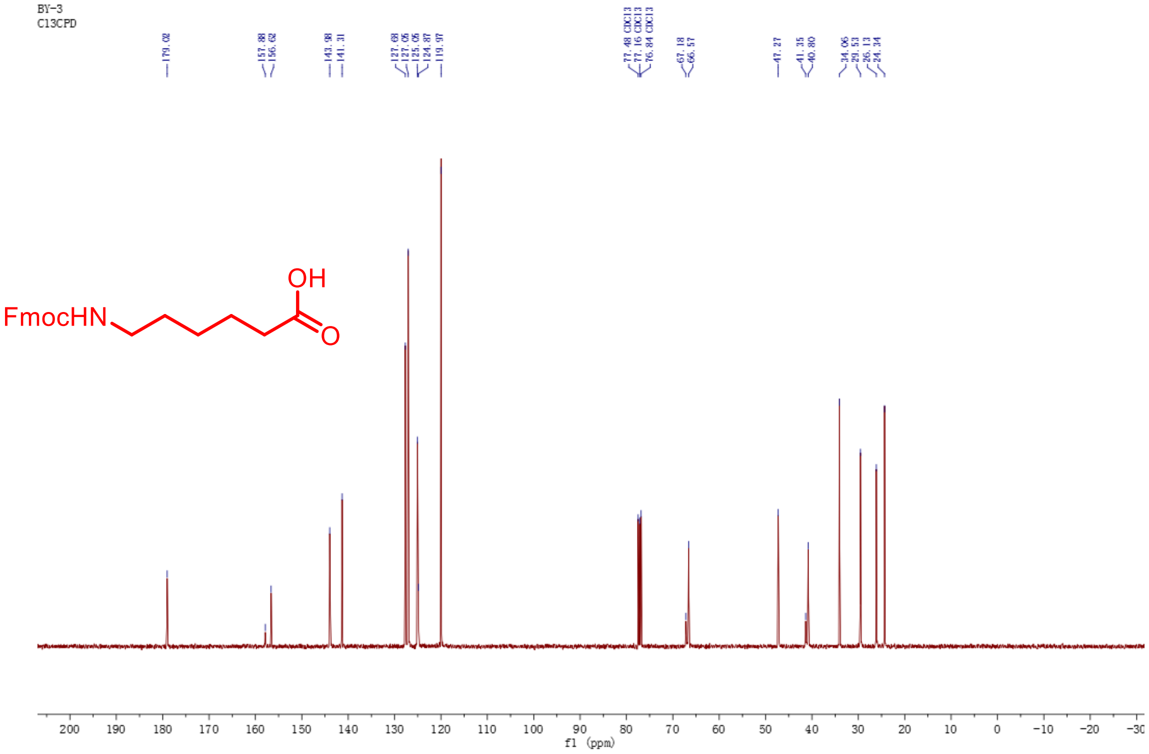
BY-3
C13CPD
FmocHN
OH
O
f1 (ppm)

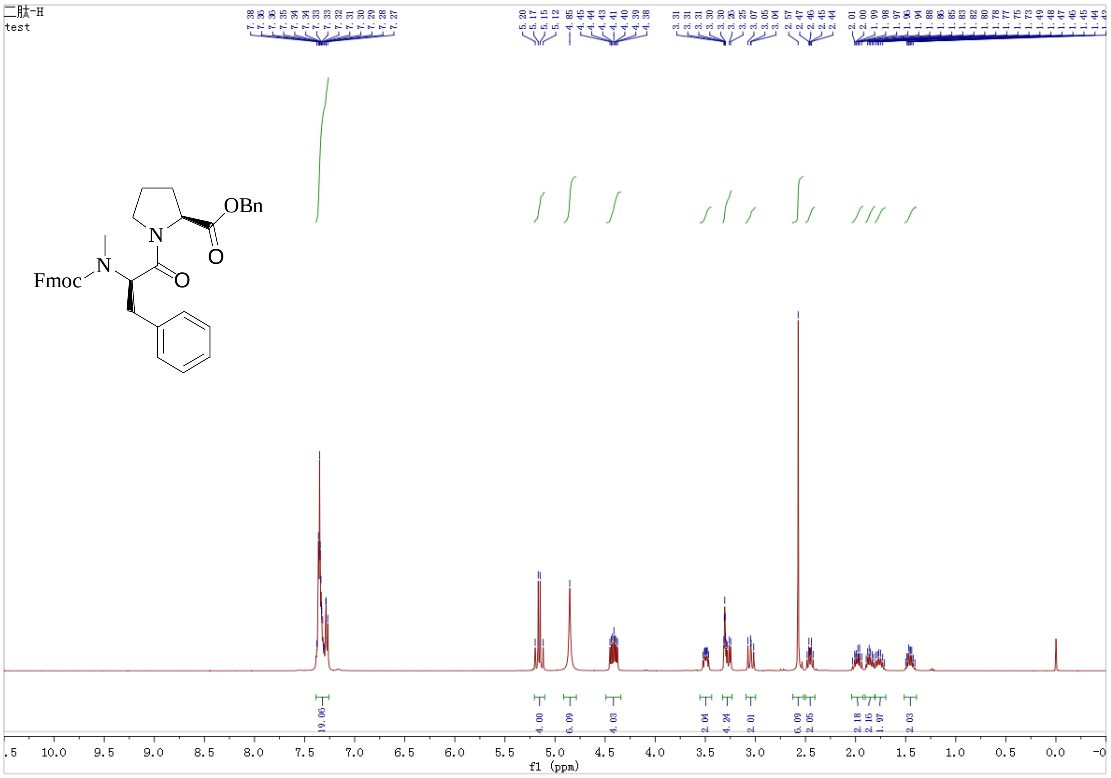

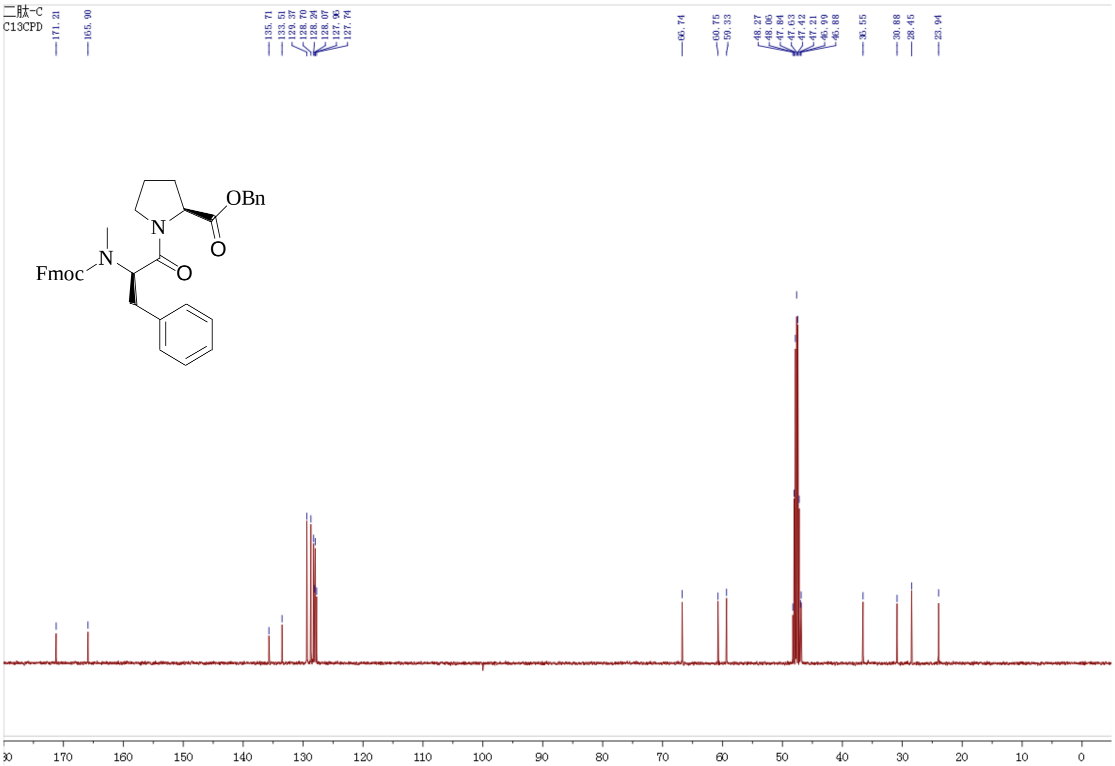

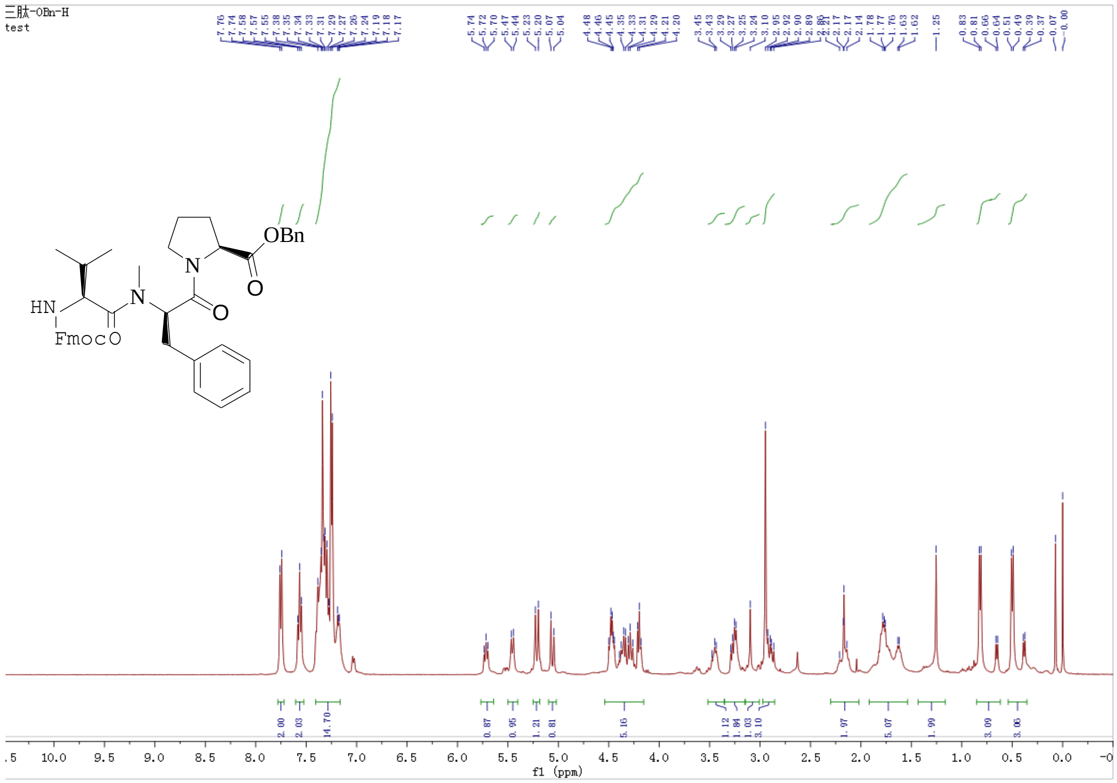

三肽-OBn-H
test
OBn
HN
Fmoc
N
O
f1 (ppm)

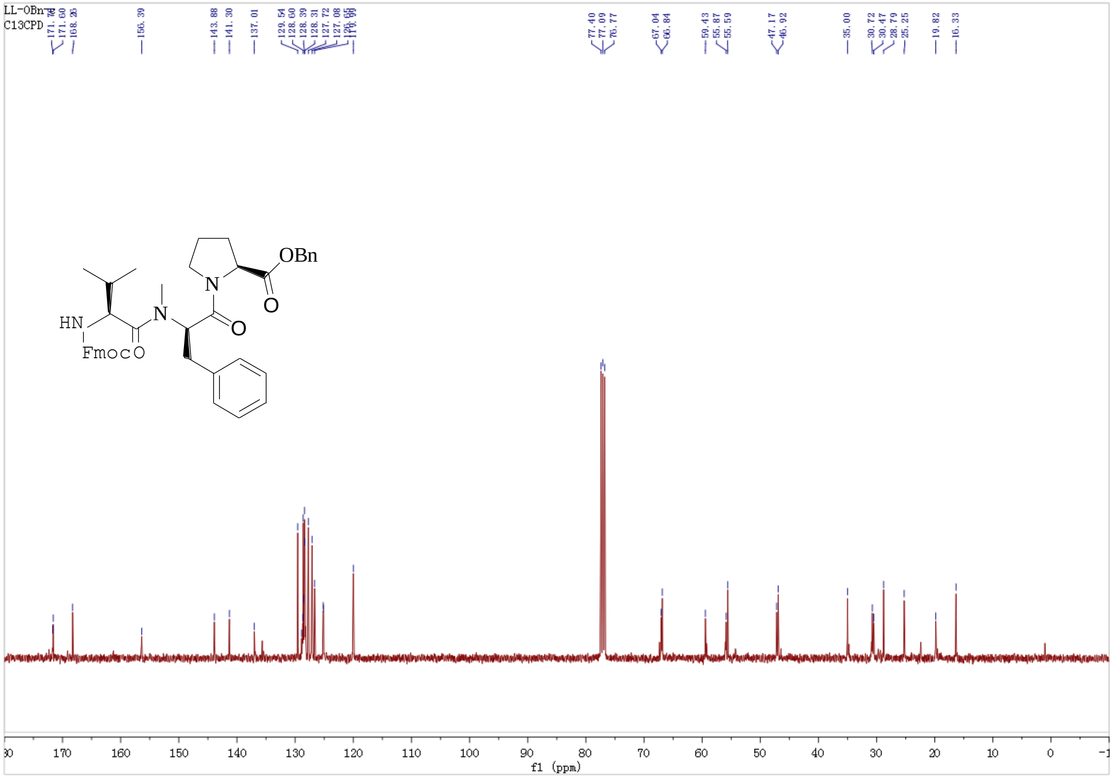

C13CPD
OBn
HN
Fmoc
N
O
f1 (ppm)

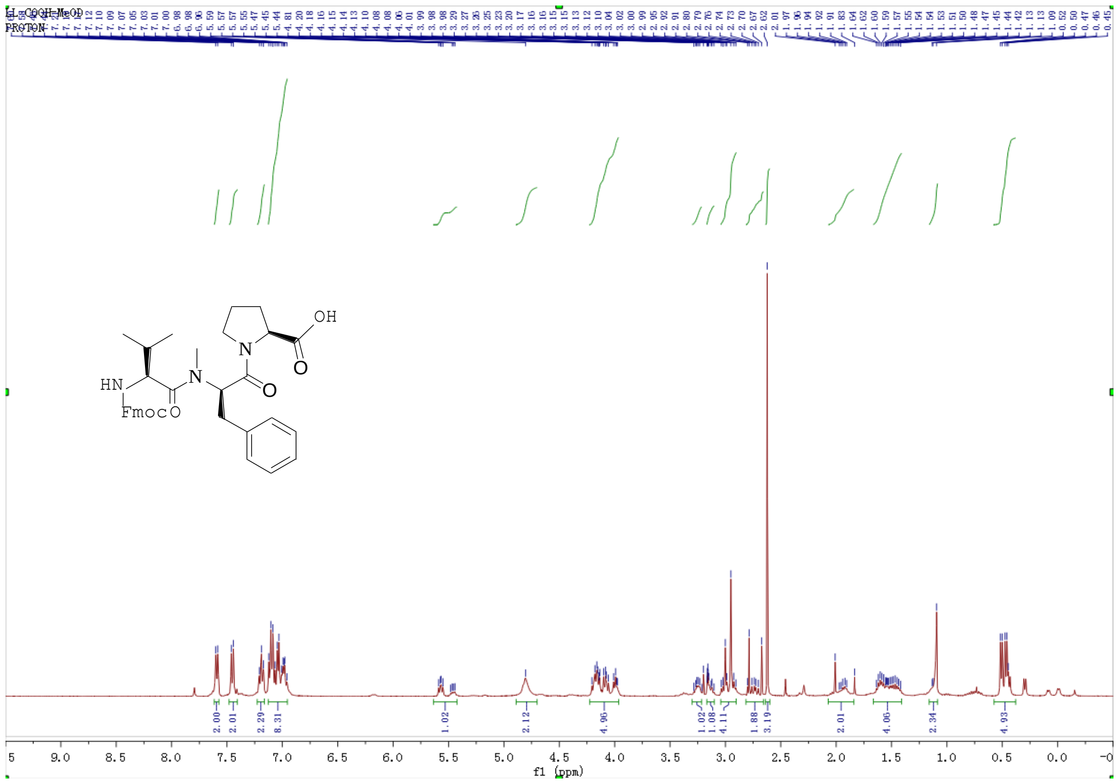
LL-COOH-MeOD
PROTON
OH
N
HN
N
O
O
Fmoc
O
f1 (ppm)

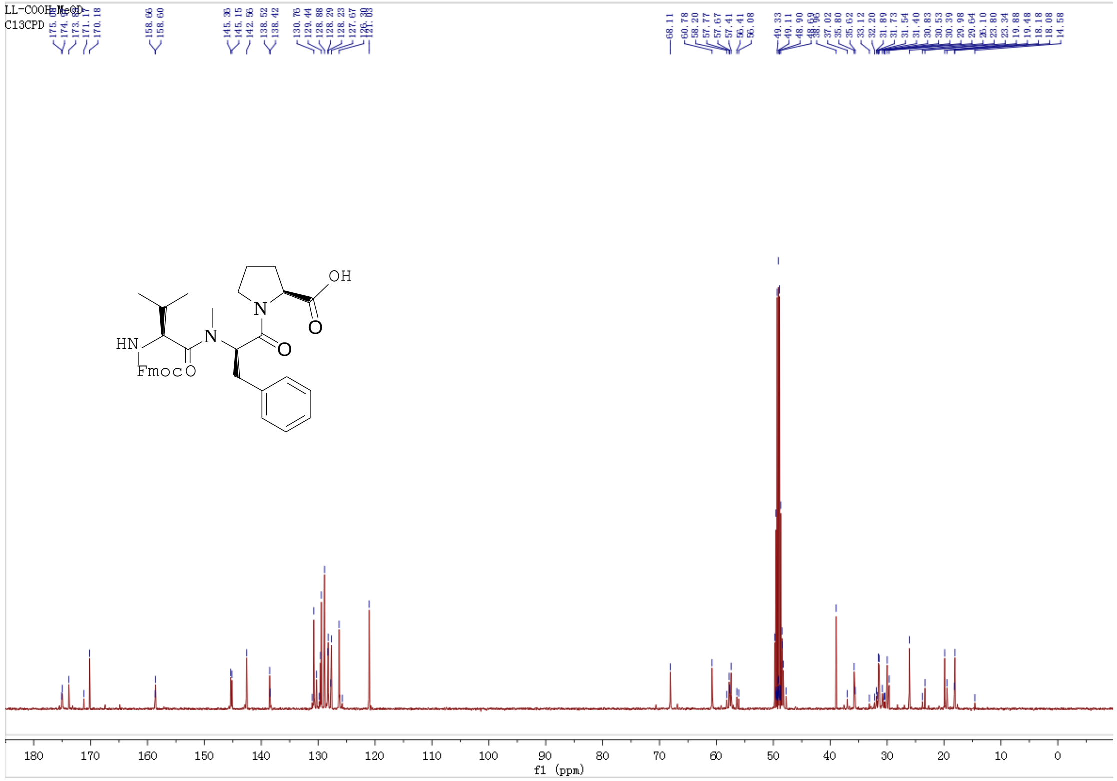
LL-COOH-MeOD
C13CPD
OH
N
HN
N
O
O
Fmoc
O
f1 (ppm)

4. ^{1}H NMR spectra, high resolution mass spectra, and analytical HPLC spectra of hoshinoamide A

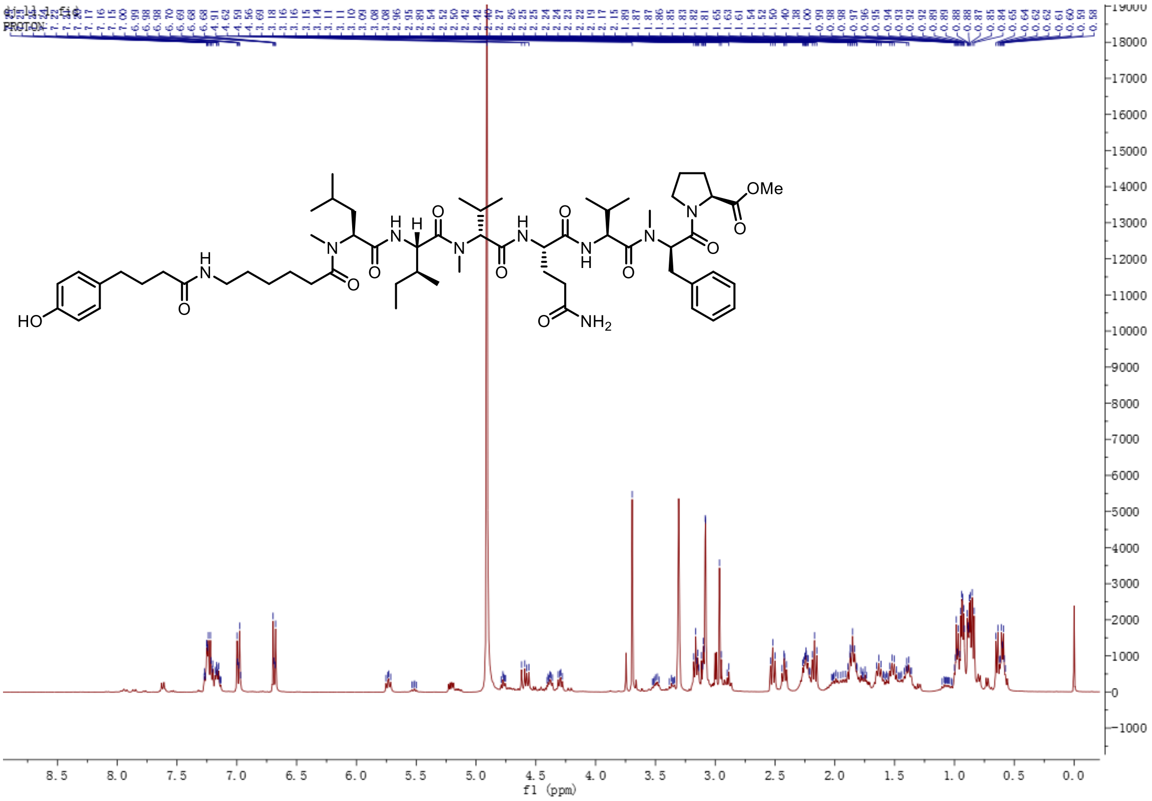

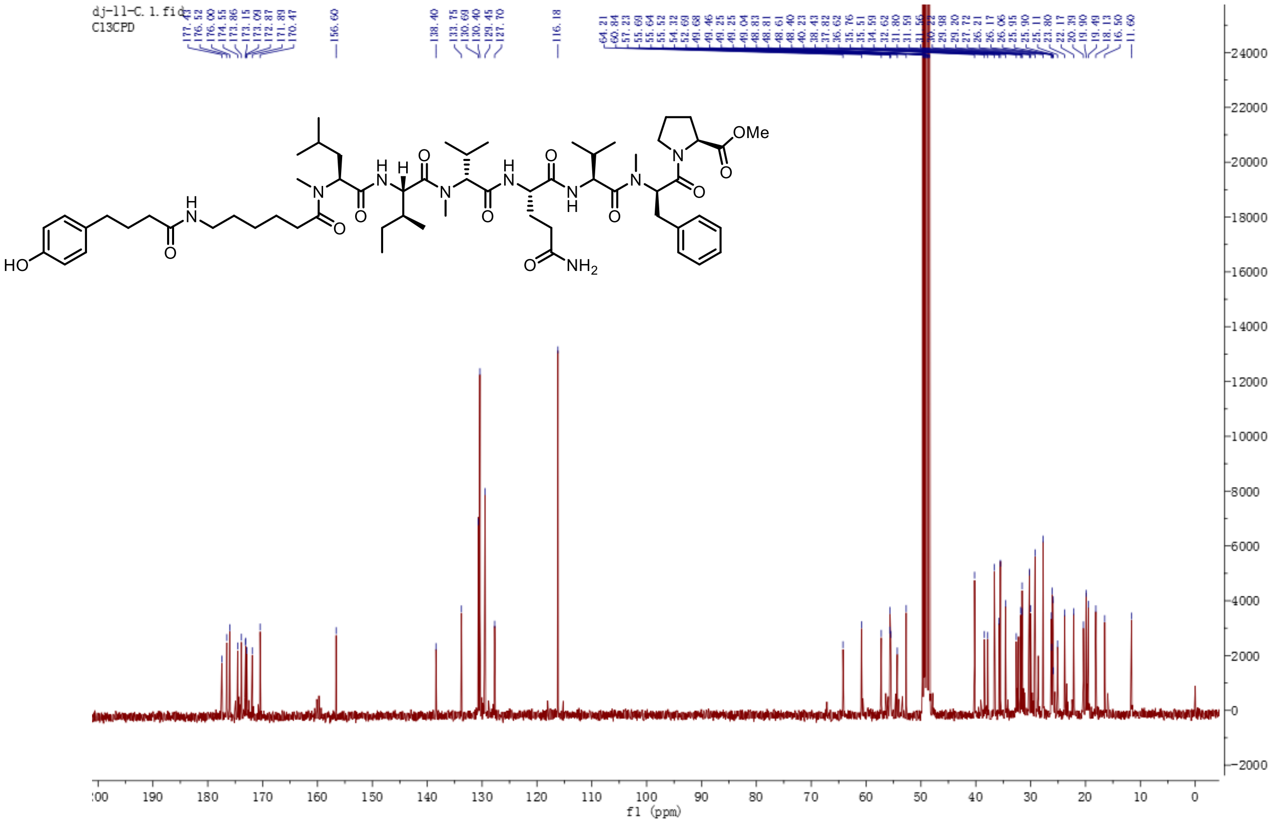

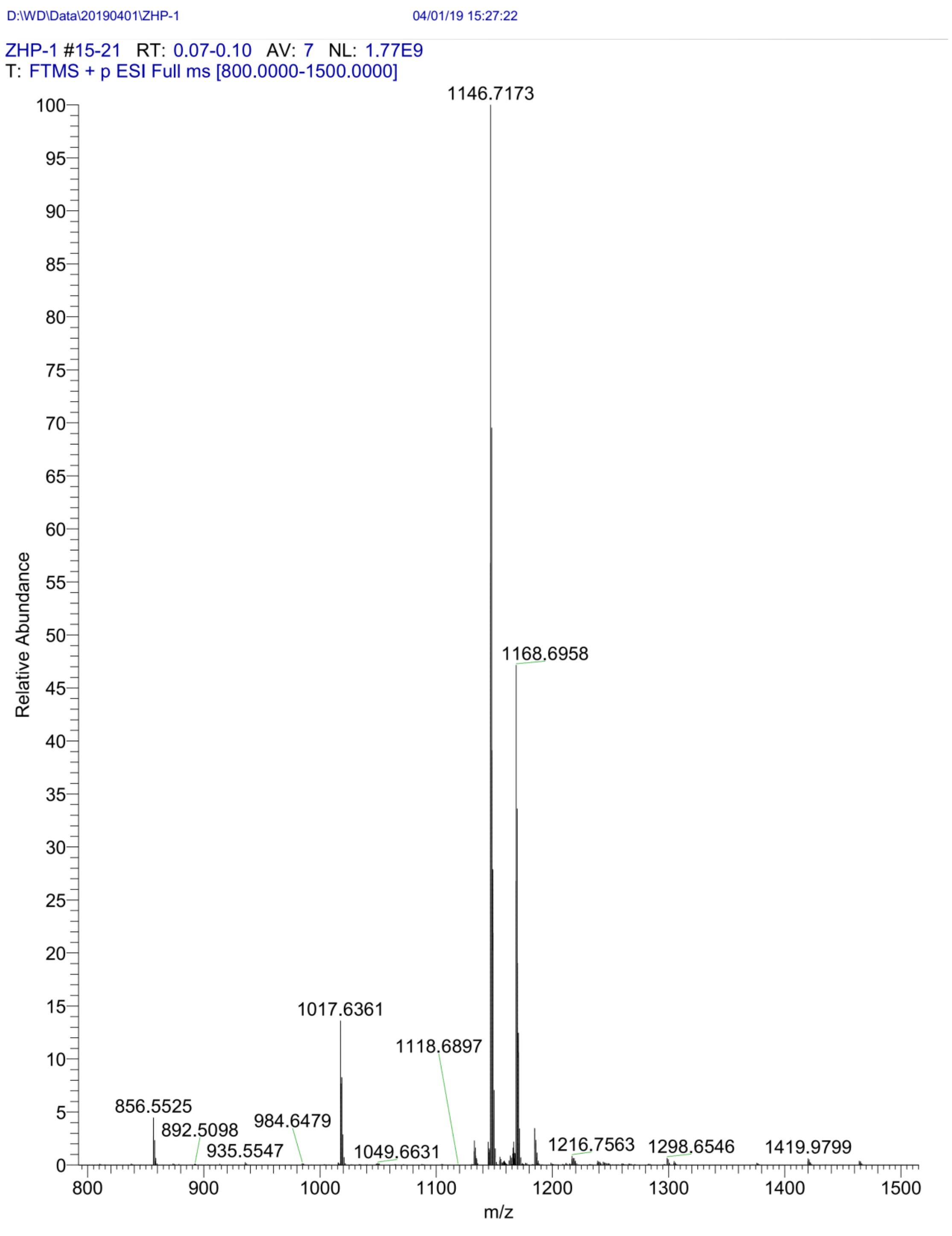

Figure S1. High resolution mass spectra of hoshinoamide A.
